# Improvement of photocatalytic degradation of organic dyes by vanadium doped titanium oxide nanoparticles using solar simulator

**DOI:** 10.1038/s41598-025-03306-y

**Published:** 2025-05-31

**Authors:** Moustafa. E. Elsisi, A. F. Mansour

**Affiliations:** Physics Department, Faculty of Science, Zagazig University, Zagazig, 44519 Egypt

**Keywords:** Organic dyes, Photocatalytic degradation, V-TiO_2_ NP_s_, Sol-gel method, Catalyst synthesis, Catalytic mechanisms, Photocatalysis

## Abstract

The removal of complex organic contaminants and pollutants from water, the heterogeneous photocatalytic degradation of methylene blue (MB), Eosin, Fluorescein and Rhodamine 6G (Rh 6G) dyes were studied using vanadium-doped titanium oxide nanoparticles (V-TiO_2_ NP_s_) as a derived catalyst, incorporated in a Polyvinyl Alcohol (PVA) solution as a host to form thin films doped from dyes and V-TiO_2_ NP_s_. Solar simulator light was tested for the photodegradation process. Vanadium-doped titanium oxide nanoparticles (V-TiO_2_ NP_s_) was successfully synthesized by a sol-gel technique and the solid-state thin films of PVA/dye/V-TiO_2_ were prepared by a casting method. The chemical synthesis of V-TiO_2_ NP_s_ was confirmed by X-ray diffractograms, Energy Dispersive X-ray (EDX), High Resolution Transmission Electron [HRTEM] and Fourier transform infrared spectroscopy [FTIR]. We had studied the effect of V-TiO_2_ NP_s_ as a catalyst to enhance the photocatalytic activity of different organic dyes and we found that the vanadium doped TiO_2_ nanoparticle behave as the best catalyst for successfully photodegraded of Methylene Blue (MB), Eosin and Fluorescein dyes but effect of V-TiO_2_ NP_s_ as a catalyst on the photodegradation of Rh 6G dye take a more time to degraded under solar simulator. The V-TiO_2_ nanoparticles displayed eminent realization for achieving nearly 99% degradation of MB, 97% of Fluorescein, 99% of Eosin after 120 min. So, the V-TiO_2_ NPs can be used as a pollutant removal in the wastewater application. But from the results of photocatalytic performance of Rh 6G, we noticed that the photodegradation of Rh 6G dye in the presence of V-TiO_2_ NPs is weak as 50% photodegraded after 300 min and this is due to the strong reaction between the catalyst and Rh 6G dye. In this case, V-TiO_2_ NPs behave as a photostabilizer for Rh 6G dye under solar simulator and this structure (Rh 6G/V-TiO_2_) can be apply as a photoanode in a dye sensitized solar cell application. The optical band gap energy of V-TiO_2_ NP_s_ with a MB, Eosin and Fluorescein dyes were calculated at zero minute and at 120 min but the band gap energy for Rh 6G dye with a catalyst was calculated at 300 min and we found that the band gap energy was increased by increasing exposure time. From the observation to measure the pseudo-first-order rate constants of four dyes were computed. The photodegradation percentage was determined and found that the photodegradation percentage of MB, Eosin and Fluorescein in the presence of the of V-TiO_2_ NP_s_ as a catalyst is higher than the photodegradation percentage of pure dyes.

## Introduction

The ever-increasing scalable utilization of organic dyes in the textile industry and their products have raised severe concerns about aquatic and non-aquatic life^1,2^. Although many attempts are underway to cope with this problem, according to environmental scientists, the threat can be mitigated through a safe photocatalytic degradation of organic dyes by utilizing easily available sunlight or ultraviolet radiation^3–5^. However, to date, photocatalytic degradation of organic dyes still lacks high efficiency^6–9^. Semiconducting metal oxides (TiO_2_, ZnO, SnO_2_, and WO_3_) are effective photocatalysts for eliminating organic and inorganic contaminants when exposed to visible or solar light due to their bandgap energy, high light absorption, and appropriate physicochemical and catalytic characteristics^10,11^.

Chemically, vanadium doped titanium oxide (V-TiO_2_) nanoparticles provide an attractive surface for tuning the interfacial characteristics and exhibit excellent blend stability. Among them, V-TiO_2_ is an inorganic semiconductor with a comparatively narrow bandgap of 3.61–2.74 eV, which is extensively investigated in various applications, such as solar transformation, photocatalysis, and photochemical hydrogen production from water. This is due to its advantages of visible-light response, ease of synthesis, low cost, and high photochemical stability^12^. Moreover, the attractive characteristics of metal oxides depend on the dispersion of cations between octahedral and tetrahedral locations within the crystal structures, controlling cation transport provides a means of tailoring their features. Cation transport depends on the ionic electrical configuration, valence, and the size of the nanoparticles^13,14^.

As a result, Vanadium is used in constructing metal oxide nanocomposites with other semiconductors to efficiently separate photo-excited electron-hole pairs and efficiently exploit the entire spectrum of sunlight, both of which are critical for photocatalytic activity. Vanadium transition metal has been effectively integrated into semiconductors, including metallic oxides^15–19^, polymers (g-C_3_N_4_)^20,21^, ternary sulfides, etc^22,23^.

Water pollution causes great damage to ecosystems, human health, as well as the sustainable economic and social development because the pollutant complex cause difficulty in decontamination by conventional water treatment processes. Hence, developing an effective and facile way to degrade pollutants has become an active area in environmental research. Recently, inorganic nanomaterials have attracted numerous attentions because of their controllable shapes and sizes, as well as their effective photocatalytic activities, such as those in metal oxide semiconductors (e.g., TiO_2_, ZnO) or narrow band gap semiconductors (e.g., Ag_3_PO_4_)^44^. Removal of toxic organic dyes from water have been provided the challenge for continued fundamental and applied research in the field of photocatalysis, synthesis of a new catalyst composite with unique nano-structure and photoelectronic properties has been received considerable interest in the recent years^45,46^. The dyes discharge in water is the foremost cause of dangerous environmental risks. Moreover, discarding toxic dyes into commonly used water allows for hazardous effect to the human and the aquatic medium. One of the most powerful applications of the nanocomposites is its photocatalytic reactivity towards the degradation of numerous toxic environmental contaminants of water. A wide spectrum of semiconductors has been used as catalysts to achieve considerable mineralization of dyes from wastewater^47^. Photocatalytic technology has shown great potential in the fields of pollutant degradation, CO_2_ reduction, hydrogen production and nitrogen fixation, furthermore photocatalysis technology is economical, toxic, and polluting-less, green, sustainable, and efficient for remediating energy and environmental issues^52^.

The vanadium doped tin oxide was achieved the highest removal efficiency of 99% degradation of MB, 97% of Fluorescein, and 99% of Eosin compared with the results of the previous research that achieved 82% a removal efficiency using the tin oxide nanoparticles with MB using UV–Visible light radiation^48^.

In this study, doping vanadium transition metal with TiO_2_ nanoparticles can enhance the overall photodegradation efficiency and photocatalytic activity of different organic dyes (MB, Fluorescein, Eosin and Rh 6G) compared to individual catalysts. Due to their high surface area, vanadium transition metal can provide more dye adsorption sites, increasing their photodegradation rate. The novelty lies in using vanadium doped titanium oxide nanoparticle, which has not been previously explored for the photodegradation of MB, Fluorescein, Eosin and Rh 6G dyes. This catalyst (V-TiO_2_) may improve performance in higher photodegradation rates and lower catalyst dosage requirements. In the present work, we had described the synthesis of unique V-TiO_2_ nanoparticles. In-depth examinations of the morphologies, structures, optical characteristics, and photocatalytic properties are conducted. The new in this paper is that we used the Fluorescein, Eosin and Rh 6G dyes with V-TiO_2_ NP_s_ to improve the photocatalytic activity of these dyes to apply and enhance the photovoltaic performance of dye sensitized solar cells. From the photocatalytic data, we observed that the Rh 6G had a low photodegradation percentage by increasing UV-irradiation time. Already, we had applied the Rh 6G dye in the dye sensitized solar cells and hybrid sensitized solar cells between dye and quantum dot to enhance the photovoltaic performance of sensitized solar cells.

## Experimental

### Materials

Titanium (IV) isopropoxide (97%, Aldrich), ethanol absolute (Adwic), Sodium Hydroxide (Aldrich), Triton -X100 (Aldrich), Acetic acid (Adwic), Ammonium Vanadate (Aldrich), Ammonium hydroxide (Aldrich), Hydrochloric acid (Adwic), Isopropanol (Adwic), Zinc Acetate (Adwic), Sodium Sulfide (Adwic), Cadmium Nitrates (Adwic), MB, Rh 6G, Eosin and Fluorescein (Dye Laser Accessories), deionized water, Ethanol absolute (99%, Bio Chem) and (Fluorine-dopped SnO_2_) conductive glass (Aldrich).

### **Synthesis of vanadium doped titanium oxide nanostructure (V-TiO**_**2**_**NP**_**s**_**)**

The Sol-gel technique was employed for the synthesis of nanostructured Vanadium doped titanium oxide nanoparticle (V-TiO_2_ NP_s_), using ammonium vanadate as the precursor. For preparing the V-TiO_2_ NP_s_, we were prepared two solutions, Solution A was prepared by mixing 40 mL of titanium isopropoxide (TIP) with 200 mL of isopropanol. Solution B was prepared by mixing 10 g of ammonium vanadate with 400 ml H_2_O + 200 ml NH_4_OH, pH adjusted by the addition of hydrochloric acid to be pH = 9 and to form one mole of vanadium transition metal in a solution. Solution A was added by drop wise to the (solution B) under rapid magnetic stirring for 4 h at 80^º^C. Finally, the solution was kept for overnight until the precipitate forms. The resultant precipitates were washed with distilled water different times to remove ammonium ions, followed by washing with ethyl alcohol more times to reduce the agglomeration of nanoparticle then drying at 60^º^C and calcination at 500^º^C^45^.

### Photocatalytic experiments

#### Preparation of a dye solution with and without V-TiO_2_ NP_s_ as a catalyst

**Fig. 1:**
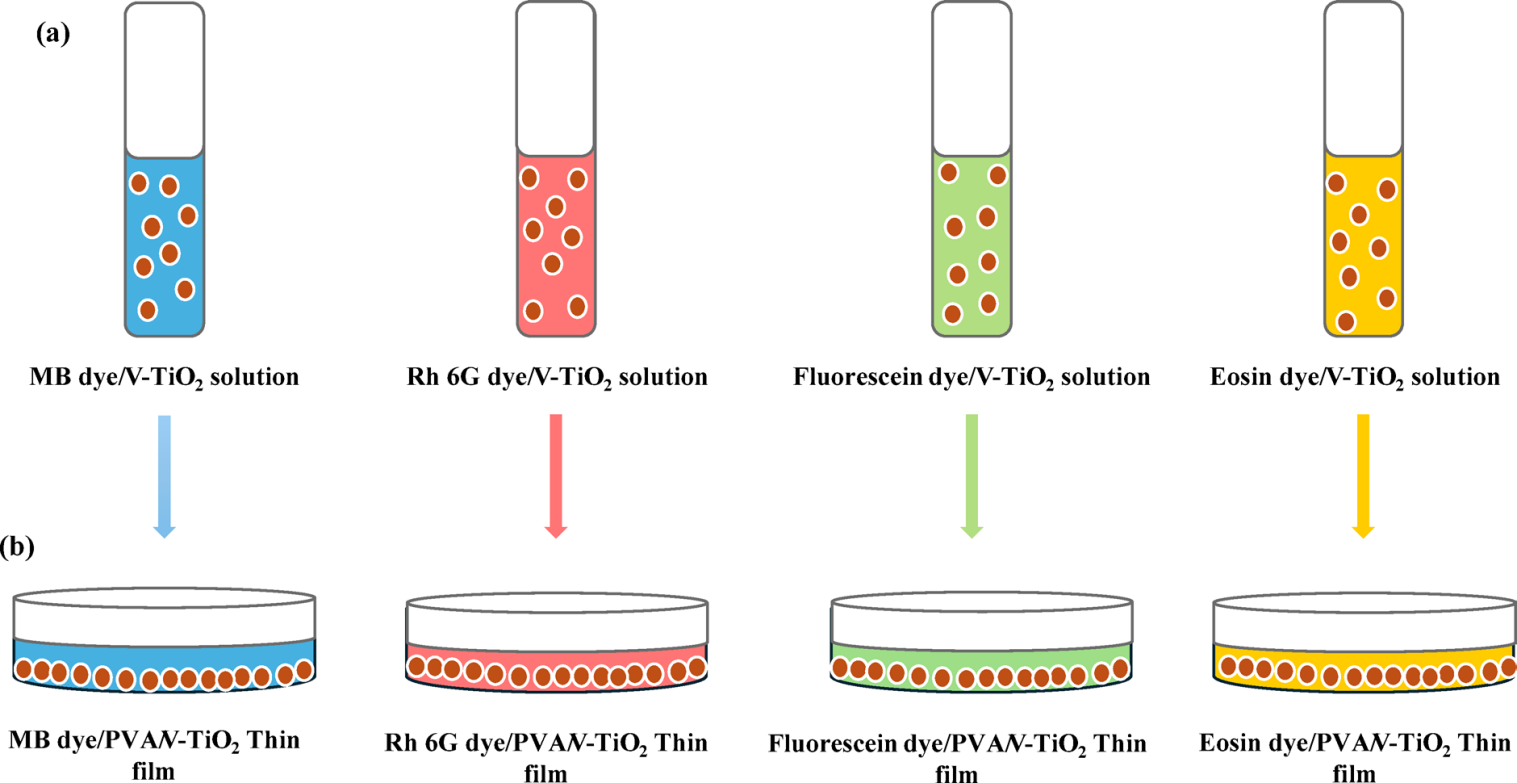
Schematic representation of Dye solution and Casting technique.

In the beginning, we had prepared a specific pure dye solution with a constant concentration 42 mg/L in a distilled water without any embedded of a V-TiO_2_ NPs catalyst for studying the photocatalytic degradation of a pure dyes (MB, Eosin, Rh 6G and Fluorescein). Secondly, As shown in Fig. 1 (a) Vanadium doped titanium oxide (V-TiO_2_) nanoparticles were assessed as a catalyst with different pollutant dyes like (MB, Eosin, Rh 6G and Fluorescein) in water with a constant concentration of 42 mg/L, using a photoreactor (solar simulator light source) (Xenon arc lamp power supply with an intensity (300 W) (Model A 6000, USA) and a fully automatic dual-range AC voltage regulator (50–130 V) (Model ST 3000 W, USA) at room temperature 25^◦^C, some factors e.g. different organic dyes and exposure time were be investigated in photocatalytic degradation process. The suspensions were stirred in the dark for 3 hr_s_. in order to establish the sorption-desorption equilibrium. For a given studied factor, 8 mL of each mixture solution of different usable dyes was putted into a quartz cuvette under continuous stirring at different exposure times (60, 120, 180, 240 and 300 min) in the front of solar simulator source. Then the mixture solution of four different organic dyes (MB, Eosin, Rh 6G and Fluorescein) in the presence of V-TiO_2_ NP_s_ as a catalyst were carried out directly into a (UV–Vis spectrophotometer 2800, United States) after each irradiation time and we had determined the optical absorbance and photodegradation percentage of a pollutant dye in the presence of a catalyst after each irradiation time by utilizing the specific absorption peaks (= 664 nm for MB, = 517 nm for Eosin, = 533 nm for Rh 6G and = 499 nm for Fluorescein)^49^.

#### Preparation of a solid-state films by doping V-TiO_2_ NP_s_ as a catalyst

As shown in Fig. 1 (b) Solid-state thin films were successfully prepared by a casting technique, using poly vinyl alcohol (PVA) as a host. Solution A: PVA solution was prepared by mixing 4 g of pure PVA with 100 mL distilled water under continuous magnetic stirring for 2 h at 50 ^o^C. Solution B: V-TiO_2_ NP_s_ as a catalyst was sonicated into a 10 mL distilled water for 5 h by using a probe sonicator. Solution B was added by drop wise to the (solution A) under magnetic stirring for another 5 h at room temperature. The full solution was divided into 4 volumes (25 mL) and in each volume we added a constant concentration of a different organic dyes (42 gm/L) as a pollutant (MB, Eosin, Rh 6G and Fluorescein). Finally, we were poured each volume in a single betray dish and leaving in air to dry then after this, we took the prepared solid-state thin films to solar simulator light for irradiating at the same different time (60, 120, 180, 240 and 300 min) and after each irradiating time, the thin films were carried out into a UV-Vis spectrophotometer. Only, we prepared solid-state thin films of PVA as a host on dye/V-TiO_2_ NP_s_ to confirm the photocatalytic degradation of organic dyes as a pollutant in water solutions and this is confirmed by disappearing the color of dye in thin films and also in water^50^.

### Methods of analysis

Different techniques were used to examine the structure and surface morphology of prepared materials.

The X-ray diffraction patterns were reported using a Pan Analytical Model X ‘Pert Pro, which was fitted with CuKα radiation (α = 0.1542 nm), Ni-filter, and a general area detector. A 40 kV accelerating voltage and a 40-mA emission current were utilized. The diffractograms were measured in the 2θ range from 0.5 to 70^o^.

The Fourier transform infrared spectroscopy (FT-IR) of the prepared samples was measured using the KBr technique adopted by the Nicolet Is-10 FT-IR spectrophotometer (Thermo Fisher Scientific. The KBr technique was conducted roughly in a quantitative manner for all samples, since the sample weight and that of KBr were both held constant.

High resolution Transmission electron microscopy (HR-TEM) images were obtained using a JEOL JEM-1230 electron microscope operating at an acceleration voltage of 120 kV for the prepared samples. The TEM measurement samples were prepared by dropping a carbon-coated copper grid with materials suspended in methanol HPLC which pre-sonicated for 15 min.

Sample optical absorption spectra were analyzed using Ultraviolet-Visible absorption spectroscopy (Spectro UV-Vis 2800, United States).

Photocatalytic Study: The V-TiO_2_ NP_s_ was applied as a photocatalyst to enhance the photodegradation of these organic dyes (MB, Eosin, Rh 6G and Fluorescein). In addition to the previous dyes were used to test the photocatalytic activity of the V-TiO_2_ NP_s_. Photocatalysis reaction was carried out in a locally made photoreactor equipped with a Xenon arc lamp power supply with an intensity (300 W) (Model A 6000, USA) and a fully automatic dual-range AC voltage regulator (50–130 V) (Model ST 3000 W, USA). The dye/V-TiO_2_ NP_s_ thin film was positioned in the front of lamp of solar simulator. The distance between the lamp and dye/V-TiO_2_ thin film is 25 cm. For this experiment, we were prepared a different thin film from PVA as a host with V-TiO_2_ NP_s_ with taking 0.15 g of V-TiO_2_ NP_s_ to dispersed in 25 mL PVA solution then loaded the different organic dyes with a constant concentration 42 mg/L on its and leaving the films to dry in air. Samples were irradiated to solar simulator light at different irradiation times and were finally analyzed by Ultraviolet-Visible absorption spectroscopy (Spectro UV-Vis 2800, United States). The percentage of photocatalytic degradation was calculated using the following Eq. [1]:

1 $$\:\text{P}\text{e}\text{r}\text{c}\text{e}\text{n}\text{t}\text{a}\text{g}\text{e}\:\text{P}\text{h}\text{o}\text{t}\text{o}\text{d}\text{e}\text{g}\text{r}\text{a}\text{d}\text{a}\text{t}\text{i}\text{o}\text{n}=\frac{\text{A}\text{o}-\text{A}}{\text{A}\text{o}}\times\:100\text{\%}\:\:$$

The rate constant of the degradation, K, was obtained from first-order plot according to the equation: ln (A_o_/A) = kt, where A_o_ is the initial absorbance of dye/V-TiO_2_ NP_s_ nanomaterial and A is the absorbance of dye/V-TiO_2_ NP_s_ under solar simulator light irradiation^24^.

## Results and discussion

### XRD analysis

**Fig. 2:**
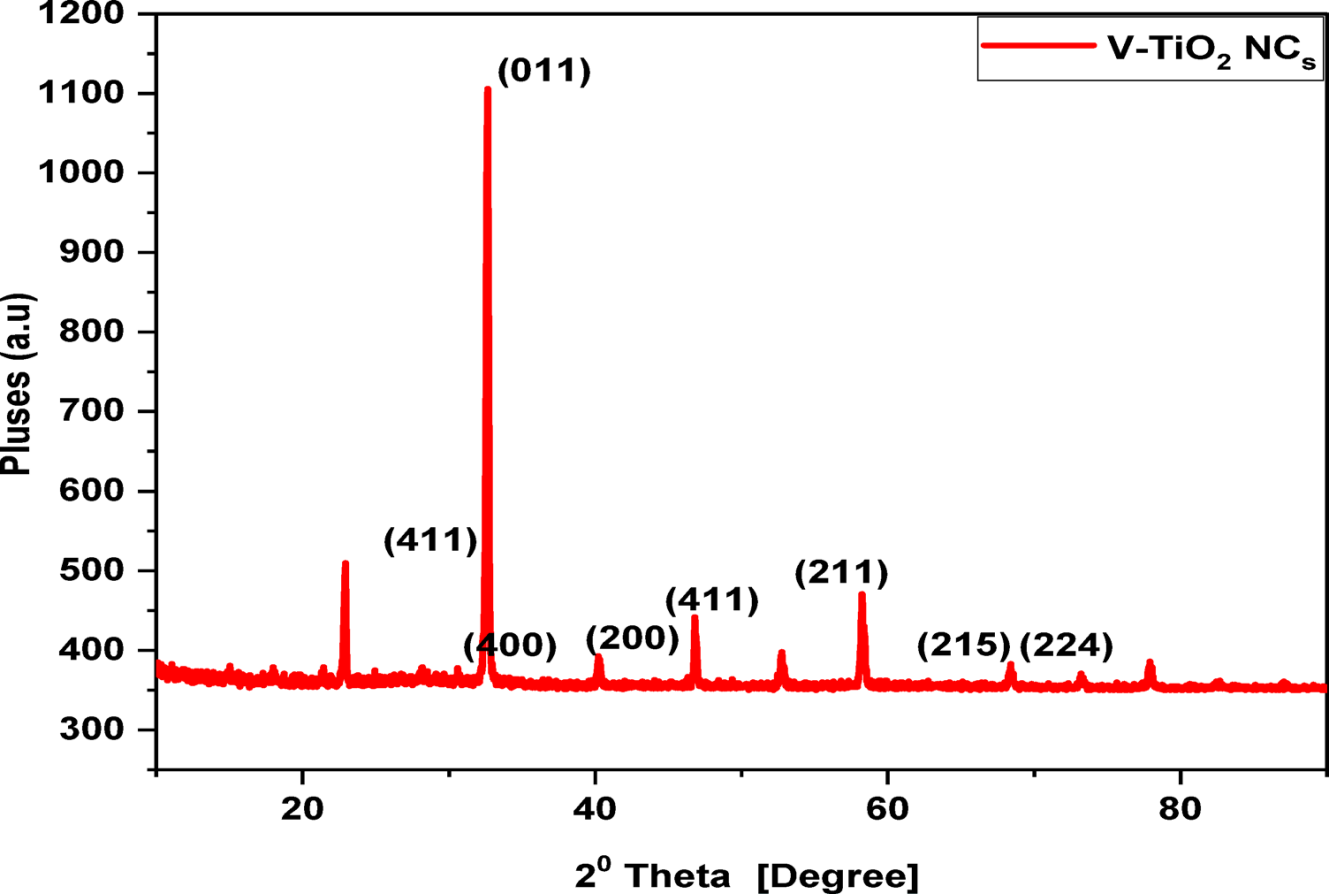
X-Ray of V-TiO_2_ NP_s_ 500^o^C.

Figure 2 shows the XRD of the prepared V-TiO_2_ NP_s_ after calcination at 500^o^C. The prepared V-TiO_2_ showed the presence of several pronounced diffraction bands at 2 θ = 22.87◦, 31.19◦, 32.61◦, 40.64◦, 45.43◦, 58.21◦, 68.80◦, 73.82◦, 75.31◦, 77.23◦, 82.53◦ and 84. 01◦. Bands at 2θ = 68.80◦, 75.31◦ and 82.53◦ correspond to the anatase phase of TiO_2_ and were assigned to (116) (215) and (224). Other phase was detected due to precipitate of vanadium oxide on titanium surface. Bands at 2 θ = 31.19◦, 32.61◦and 45.43◦ were attributed to V_2_O_5_^25^. The observed bands correspond to (400), (011) and (411). Other bands at 2θ = 40.64◦, 58.21◦ and 73.82◦ were attributed to Ti-V and were assigned to (200) and (211).

### FTIR analysis

**Fig. 3:**
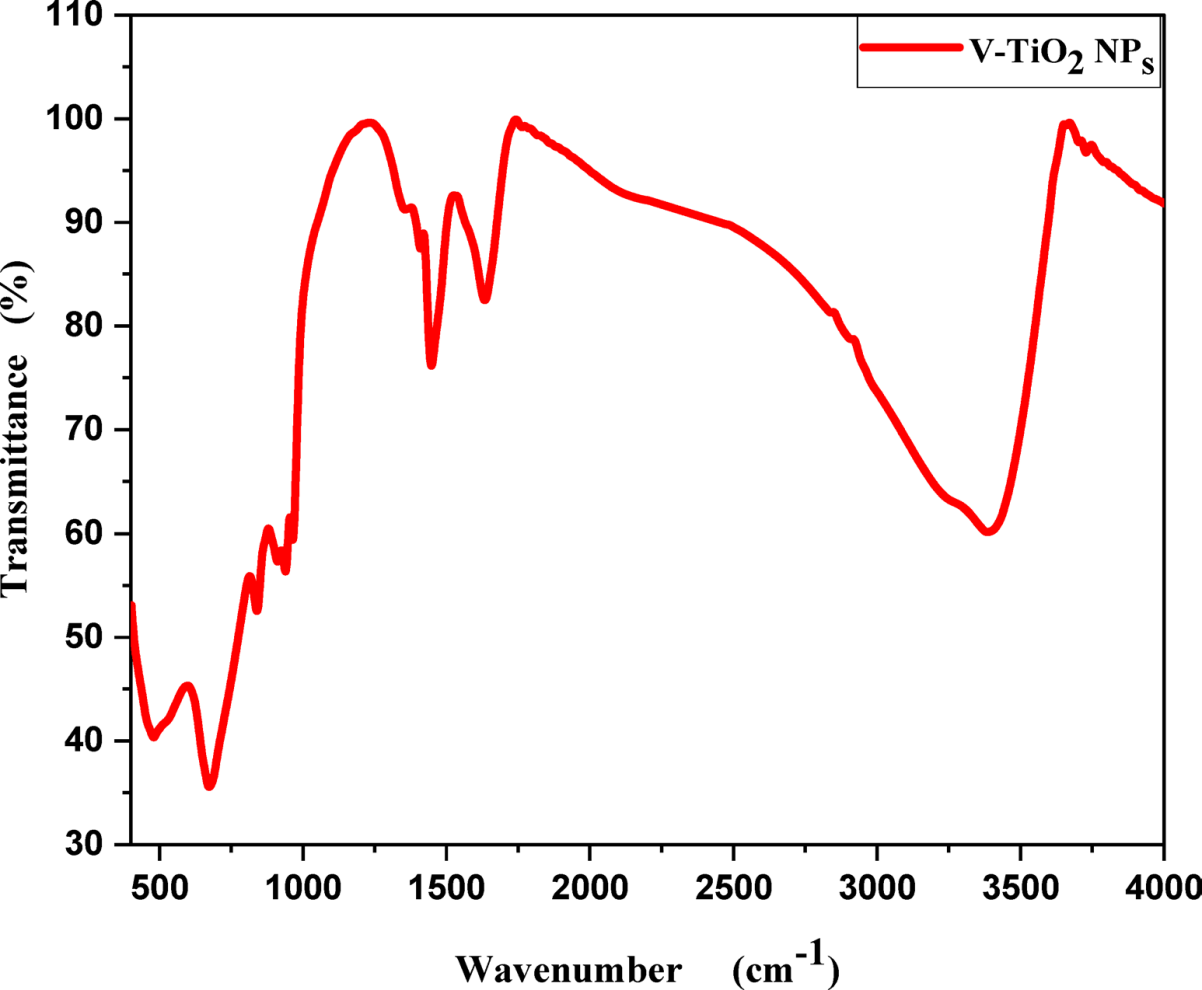
FTIR spectra of V-TiO_2_ NP_s_ at 500^o^C.

Figure 3 shows FTIR spectra of the V-TiO_2_ NP_s_ annealed at 500^o^C. The absorption peak at 1625 cm^−1^ could be attributed to hydroxyl (bending) groups of molecular water. A broad peak at 1401 cm^−1^ could be attributed to the hydrated vanadium oxides^26^. Vanadate group has characteristic frequency between 800 and 1000 cm^−1^, the absorption peaks at 899 cm^−1^ and 869 cm^−1^ could be attributed to V-O-V^27–30^. Peak at 987 cm^−1^ could be assigned to V = O^31^. Absorption Peak at 686 cm^−1^ could be ascribed to Ti–O–V^32,33^ and the peak around 500 cm^−1^ could be attributed to symmetric V-O-V^34^. Peak at 708 cm^−1^ could be assigned to Ti–O–V. peaks which belong to vanadium group observed at 981 cm^−1^,899 cm^−1^ and 755 cm^−1^ which attributed to V = O, V-O-V and asymmetric stretch V-O-V^35^ with New peak observed at 464 cm^−1^ that may be attributed to symmetric stretch V-O-V^34^.

### Raman spectroscopy

**Fig. 4:**
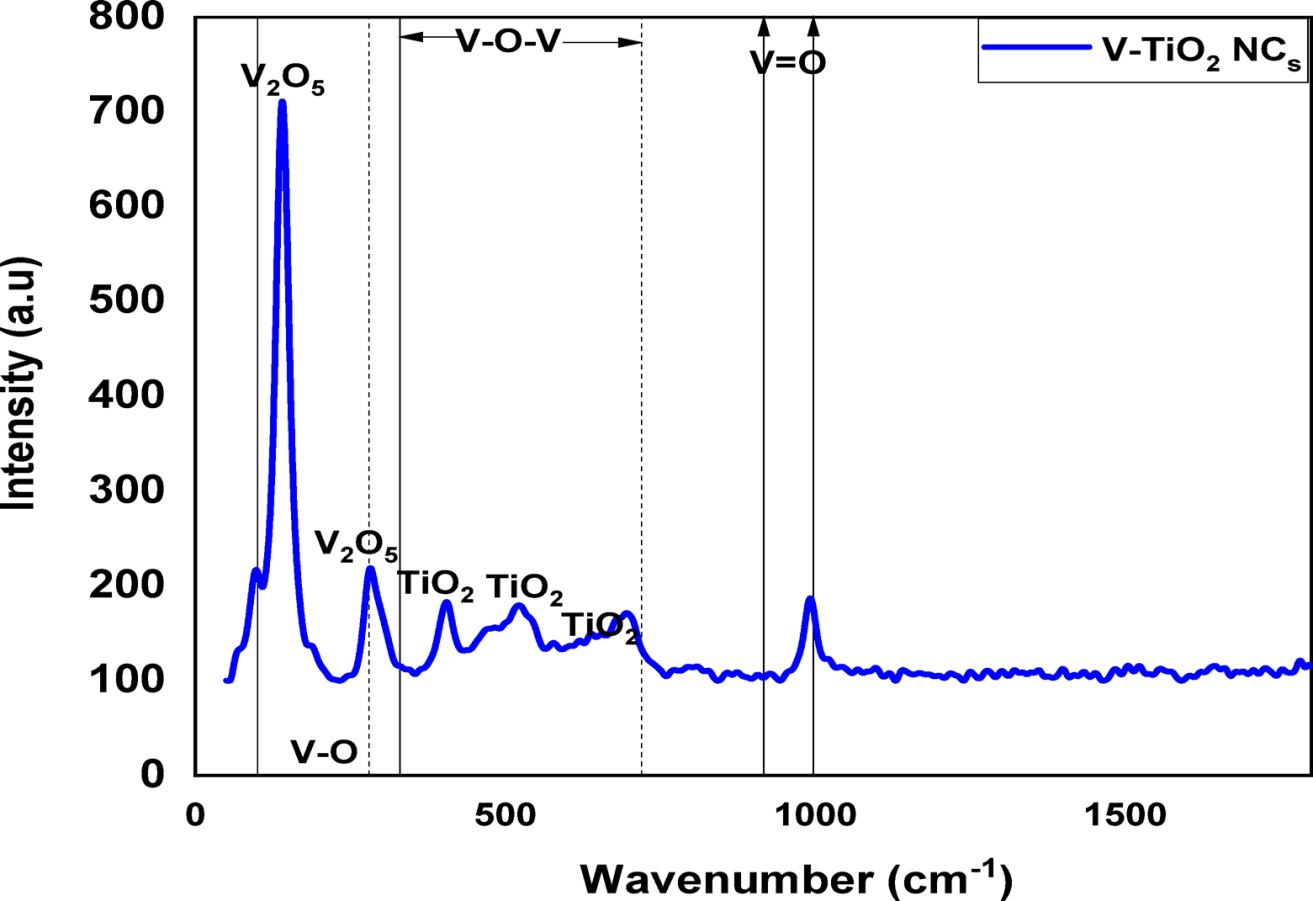
Raman of V-TiO_2_ NP_s_ at 500 ^o^C.

Figure 4 shows Raman spectra of the V-TiO_2_ after annealed at 500 ^o^C. The several peaks around (110, 140, 282, 319,405, 479, 512, 578, 632, 695,917,997 and 1033 cm^−1^) were observed. Peaks at 405,512,632 cm^−1^ correspond to the anatase phase of TiO_2_.Other peaks at 110,140, 282,319, 479, 578,695,719 and 997 cm^−1^ correspond to V_2_O_5_^36,37^. Peaks in the range of 100–330 cm^−1^ are related to the oxides of V modes and are mostly from the lattice and bending modes of V–O.

Peaks in the range of 280 cm^–1^ to720 cm^−1^ could be attributed to V-O-V stretching and bending modes. The peak located at 917,997 cm^−l^, indicates the symmetric V = O stretching mode. We notice that the peak at ~ 1030 cm^−1^ has been also attributed to monomeric vanadyl (V^4+^) species bound directly to the TiO_2_ support^38^.

### EDX analysis

**Fig. 5:**
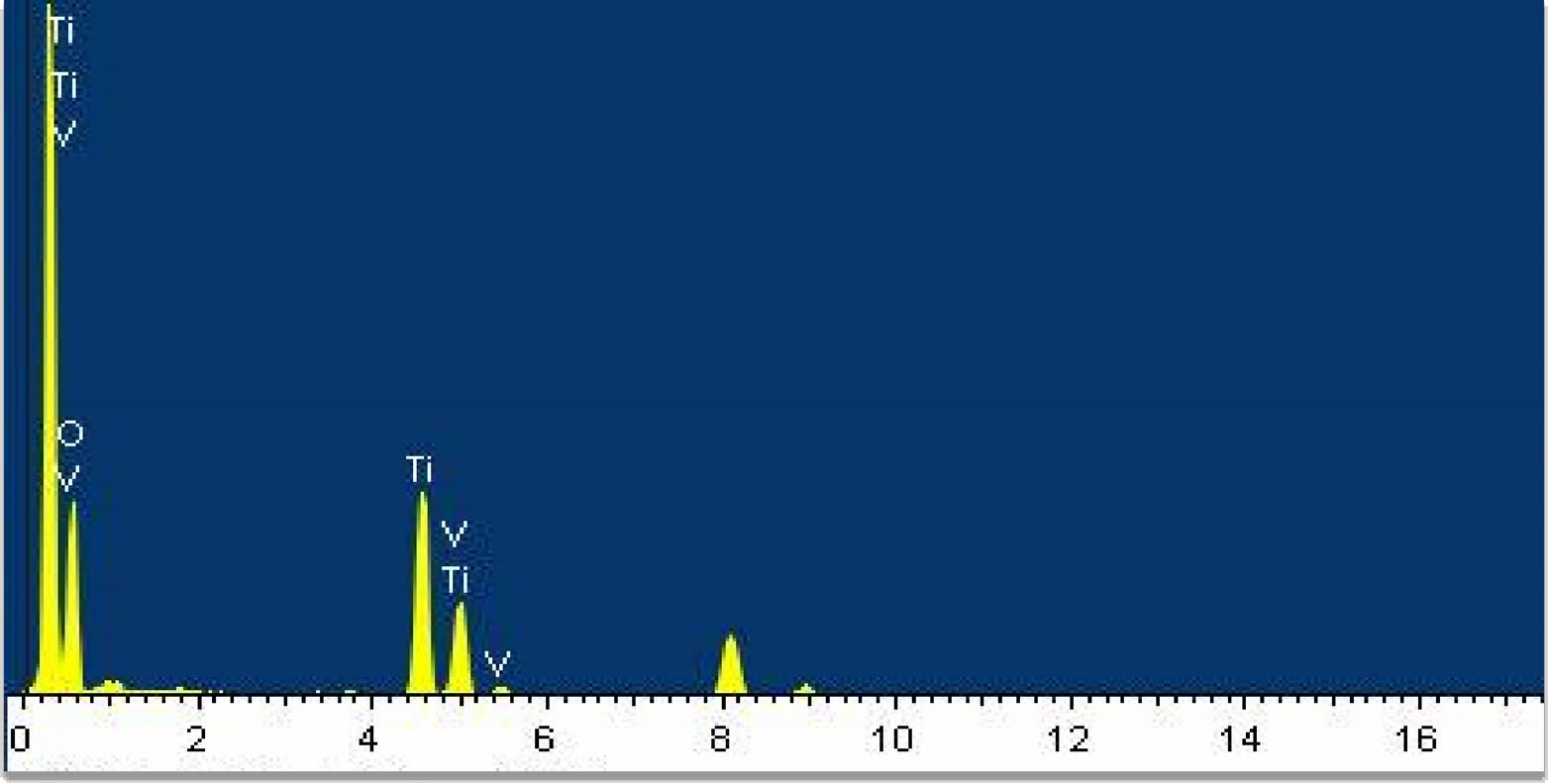
EDX of the V-TiO_2_ NP_s_ at 500 ^o^C.

Figure 5 shows EDX spectrum of the prepared V-TiO_2_ NP_s_ annealed at 500 ^o^C. The figure shows that No elements other than V, O and Ti are present in EDX; it certifies the purity of the prepared samples. Atomic constituents and weight% of V, O and Ti were observed in Table 1.

**Table 1:** Element constituents, weight% and atomic percentage of V-TiO_2_ NPs.

Element	Weight% (%)	Atomic weight% (%)
Ti	60.42	41.25
V	23.5	30.85
O	16.08	27.9

### HR-TEM

**Fig. 6:**
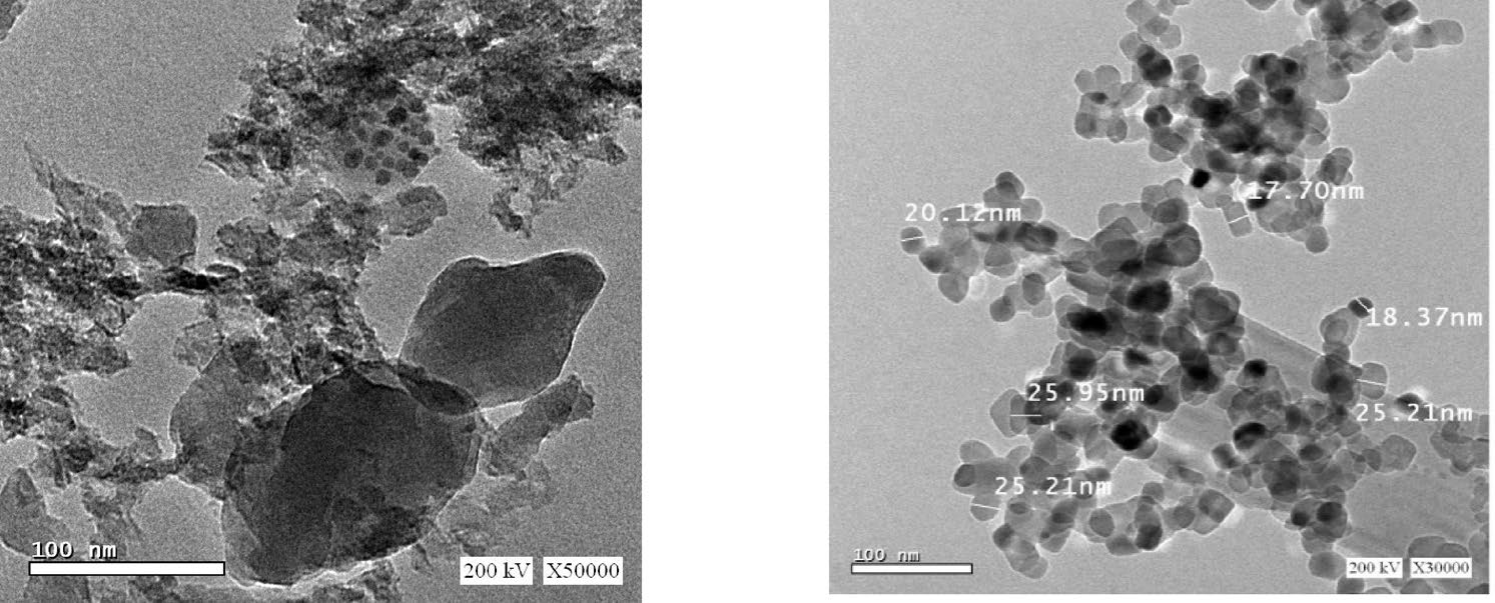
HRTEM of V-TiO_2_ NP_s_ at 500 ^o^C.

Figure 6 shows high resolution transmission electron microscopy (HRTEM) of the V-TiO_2_ NP_s_ calcined at 500^o^C. After calcination to 500^o^C the particle size distribution assembled to aggregates; the crystallite size ranged 17–26 nm which in good agreement with the results of XRD obtained by Debye-Sherrer formula.

### Thermogravimetric analysis (TGA)

**Fig. 7:**
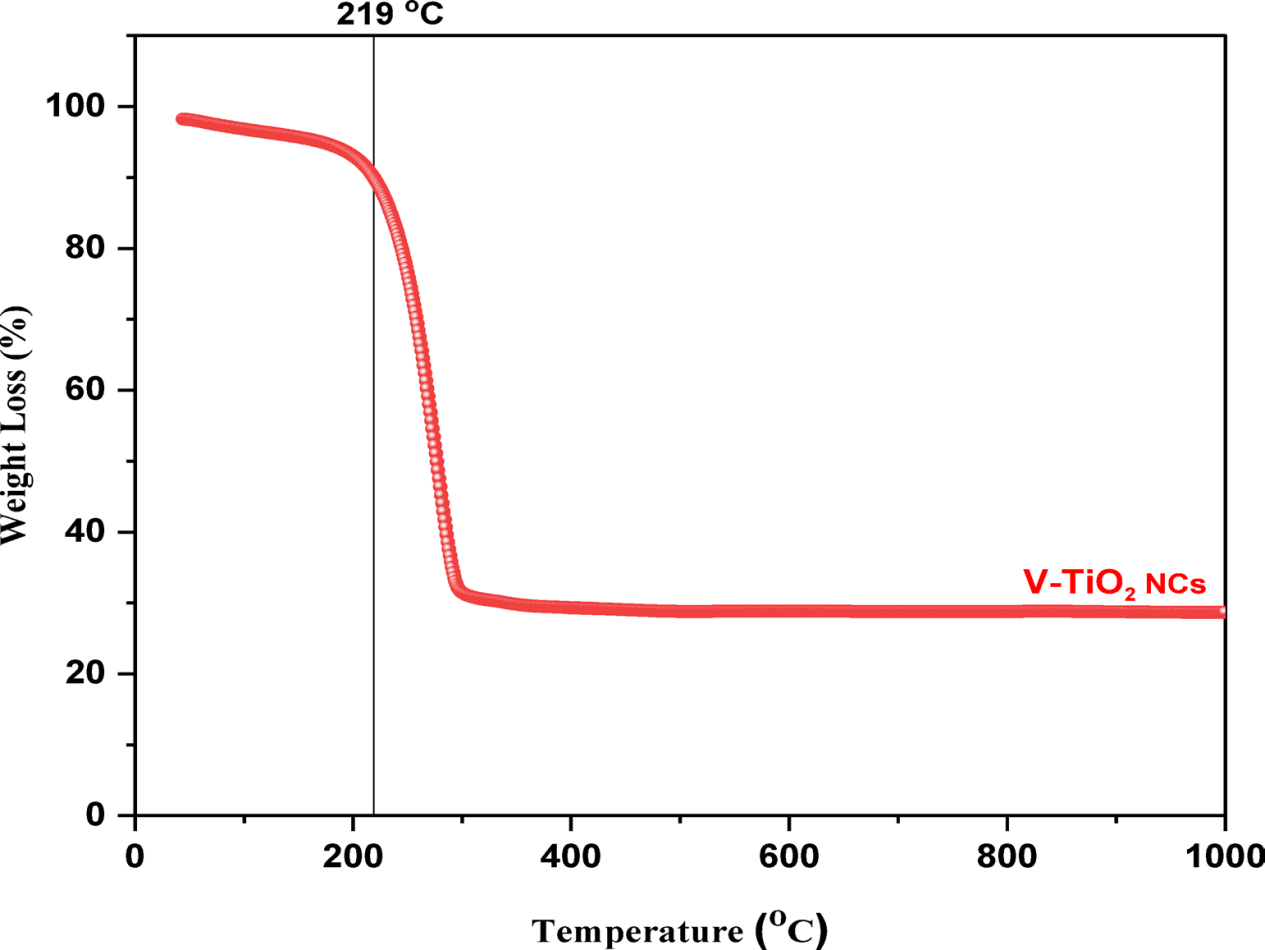
TGA of V-TiO_2_ NP_s_.

Figure 7 shows Thermogravimetric analysis (TGA). The thermal stability of the prepared V-TiO_2_ NP_s_ was measured from room temperature to 600^o^C in air at heating rate of 6^o^C/min. Evidently, the weight loss proceeded early in stages with increasing temperature, while the most significant weight loss occurred before 250^o^C due to the volatilization and thermal decomposition of the remaining organics. After 250^o^C the thermogravimetric curve showed a stable curve without significant loss in weigh characteristic and loss curve levels off.

### Optical band gap energy of pure V-TiO_2_ NPs without loading dyes and with immersed in MB, Eosin, fluorescein and Rh 6G dyes at different irradiation times

**Fig. 8:**
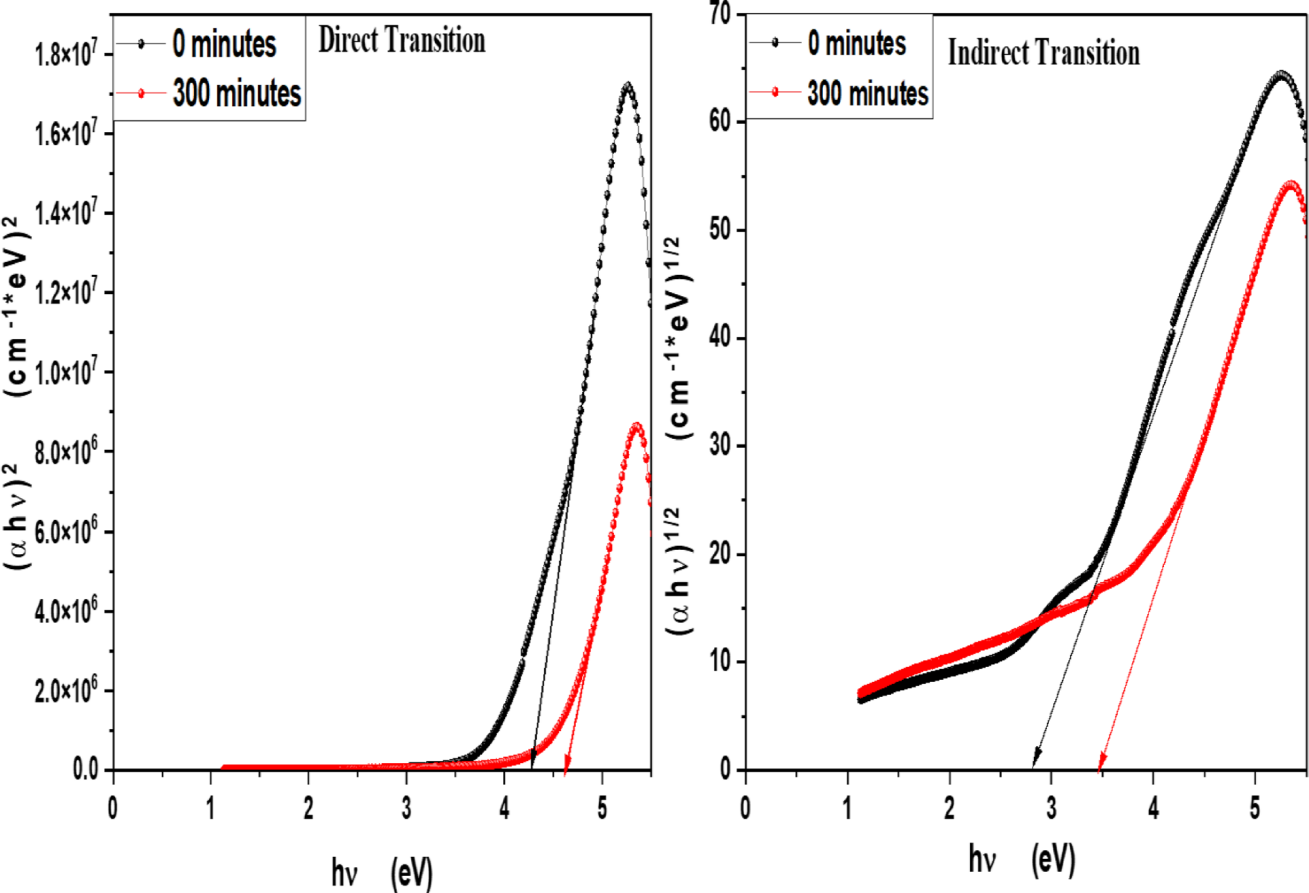
Tauc Plot of pure V-TiO_2_ NP_s_ before and after exposure to light for direct transition and indirect transition.

**Fig. 9:**
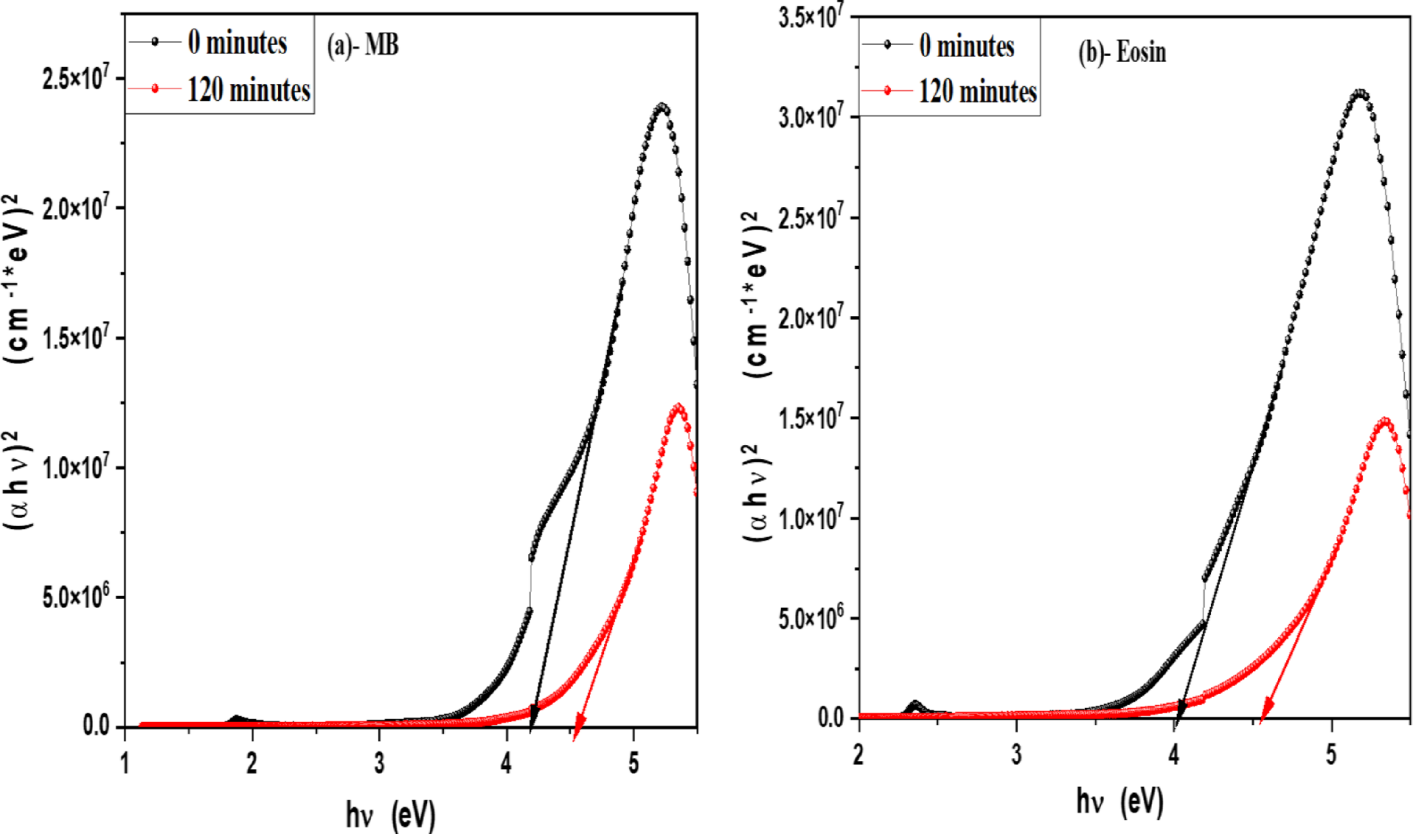
Plot of (αhʋ)^2^ versus hʋ for (**a**)- MB dye and (**b**)- Eosin in the presence of the V-TiO_2_ NP_s_ before and after exposure to light for direct transition.

**Fig. 10:**
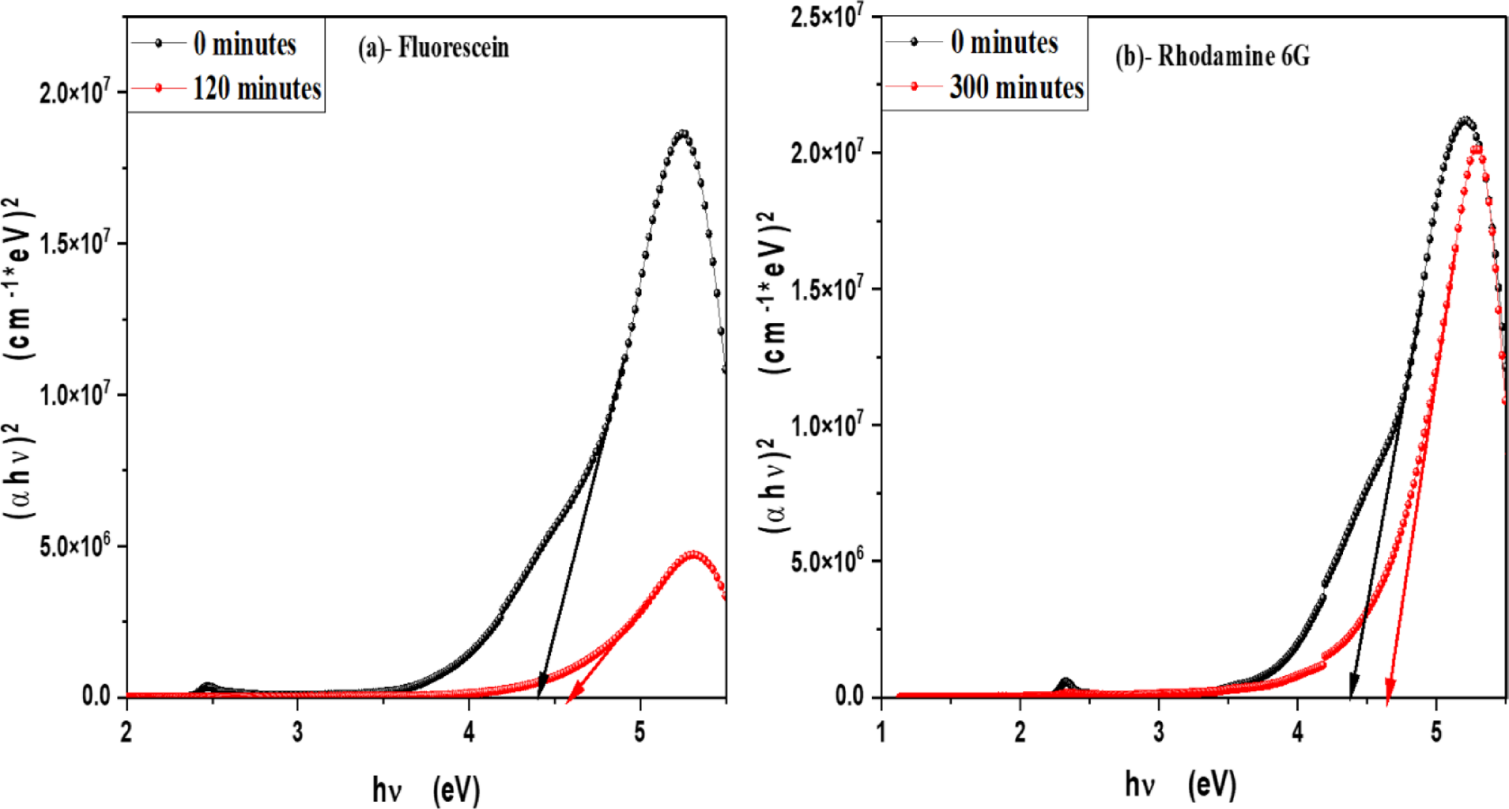
Plot of (αhʋ)^2^ versus hʋ for (**a**)- Fluorescein and (**b**)- Rhodamine 6G dyes in the presence of the V-TiO_2_ NP_s_ before and after exposure to light for direct transition.

**Fig. 11:**
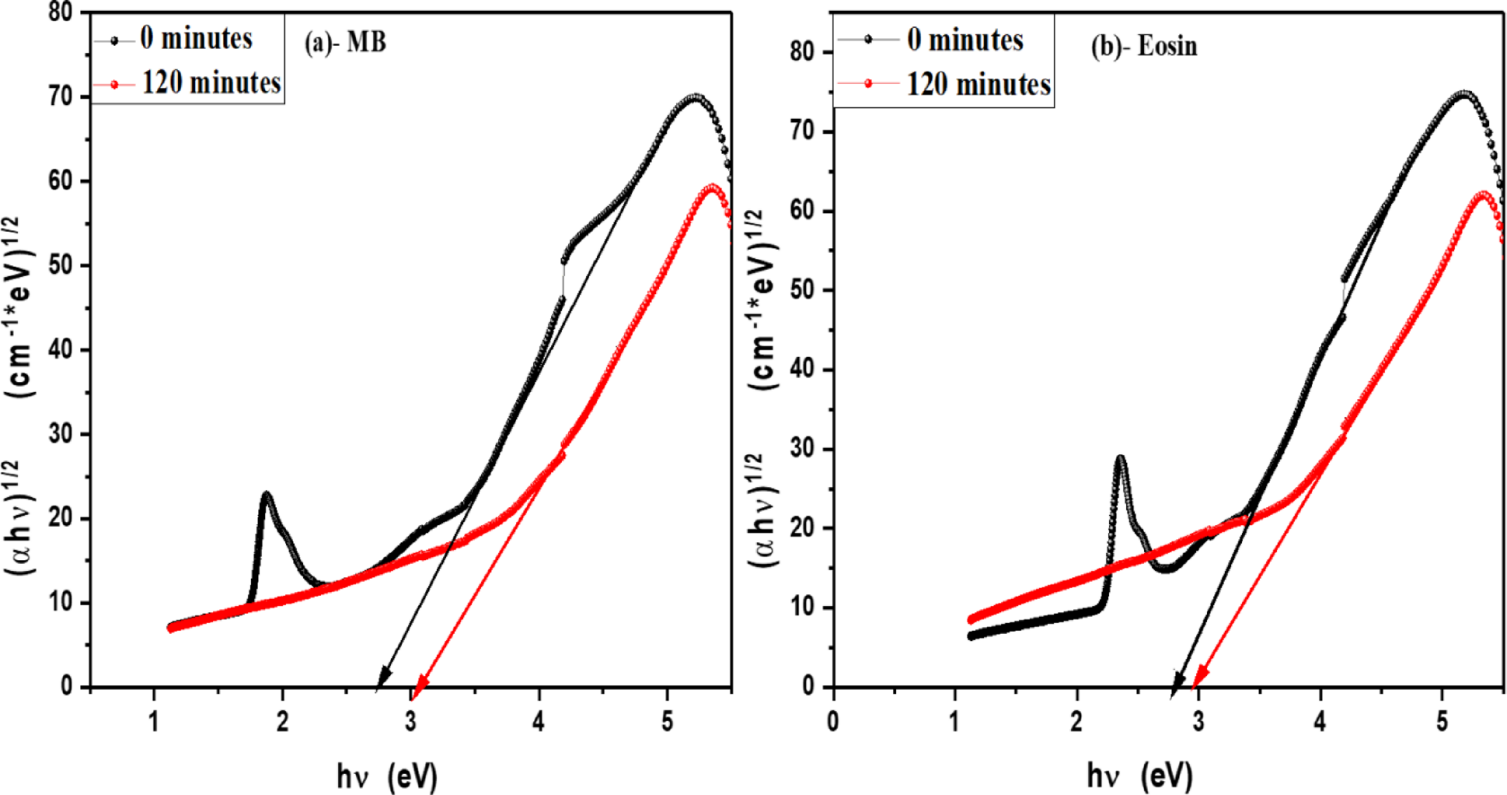
Plot of (αhʋ)^1/2^ versus hʋ for (**a**)- MB dye and (**b**)- Eosin in the presence of the V-TiO_2_ NP_s_ before and after exposure to light for indirect transition.

**Fig. 12:**
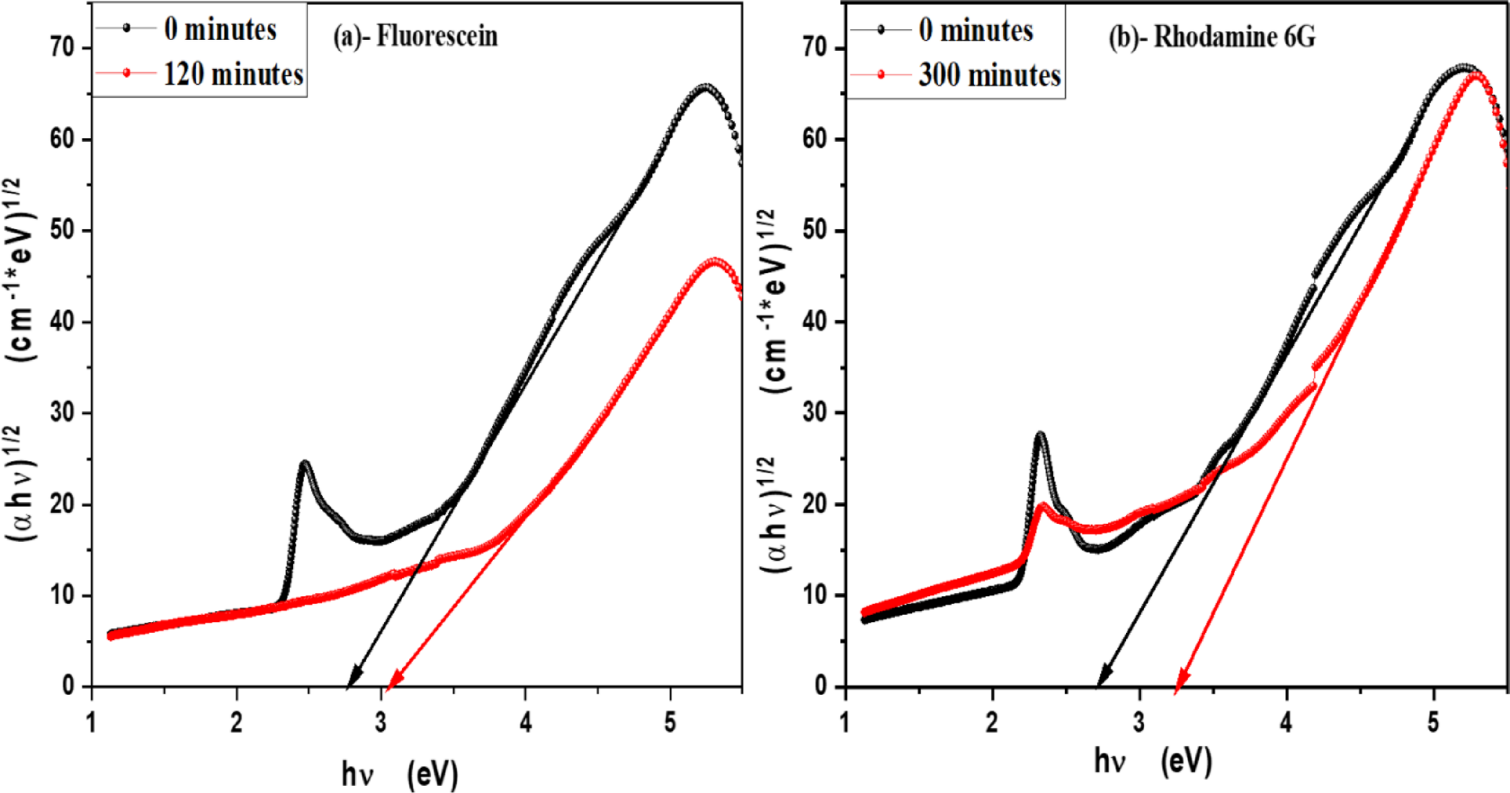
Plot of (αhʋ)^1/2^ versus hʋ for (**a**)- Fluorescein and (**b**)- Rhodamine 6G dyes in the presence of the V-TiO_2_ NP_s_ before and after exposure to light for indirect transition.

In order to determine the optical band gap, we used the Tauc formula, which is as follows: (αhv) = A (hυ - Eg)^n^, where hυ is the photon energy, E_g_ is the energy gap, and n is the quality of the transitions. For a direct transition, the term n is used as 2, whereas for an indirect transition, it is ½. Plotting (αhυ)^1/n^ versus photon energy and tangent-drawing the curve that meets the energy axis at α = 0 are the methods used to examine the optical bandgap energy. Figure 8 shows optical band gap energy of pure V-TiO_2_ NP_s_ in both direct and indirect transition and shows that the optical band gap energy of pure V-TiO_2_ NP_s_ is decreasing by increasing irradiation time that shown in Table 2. For direct permitted transition, Figure 9 (a&b) displays the Tauc plot of MB and Eosin dye in the presence of the catalyst V-TiO_2_ NP_s_. Figure 10 (a&b) displays the Tauc plot for Rh 6G and Fluorescein dye in the presence of the catalyst V-TiO_2_ NP_s_ that have a direct allowed transition. Also, Figure 11 (a&b) and Figure 12 (a&b) shows the Tauc plot for indirect allowed transition for MB and Eosin dye in the presence of V-TiO_2_ NP_s_, Rh 6G and Fluorescein dye in the presence of V-TiO_2_ NP_s_ respectively. All of the Tauc plots show that the optical band gap energy of V-TiO_2_ NP_s_ immersed in dyes increasing by increasing irradiation exposure time that are shown in Table 3.

**Table 2:** Optical band gap energy of pure V-TiO_2_ NP_s_ before and after irradiation for direct and indirect transition.

Catalyst	Direct Transition		Indirect Transition	
V-TiO_2_ NPs	0 min	5 min	0 min	5 min
	4.27 eV	4.61 eV	2.82 eV	3.47 eV

**Table 3:** Optical band gap energy for MB, Eosin, Rh 6G and fluorescein dyes in the presence of V-TiO_2_ NP_s_ for direct and indirect transition.

Catalyst	Dyes	Exposure Time (hr)	E_g_ for Direct transition	E_g_ for indirect transition
V-TiO_2_ NP_s_	Methylene Blue	0 min	4.21	2.75
		120 min	4.56	3.07
	Eosin	0 min	4.38	2.8
		120 min	4.66	3.26
	Fluorescein	0 min	4.41	2.78
		120 min	4.59	3.07
	Rhodamine 6G	0 min	4.02	2.74
		300 min	4.56	2.96

### Photocatalytic performance evaluation

**Fig. 13:**
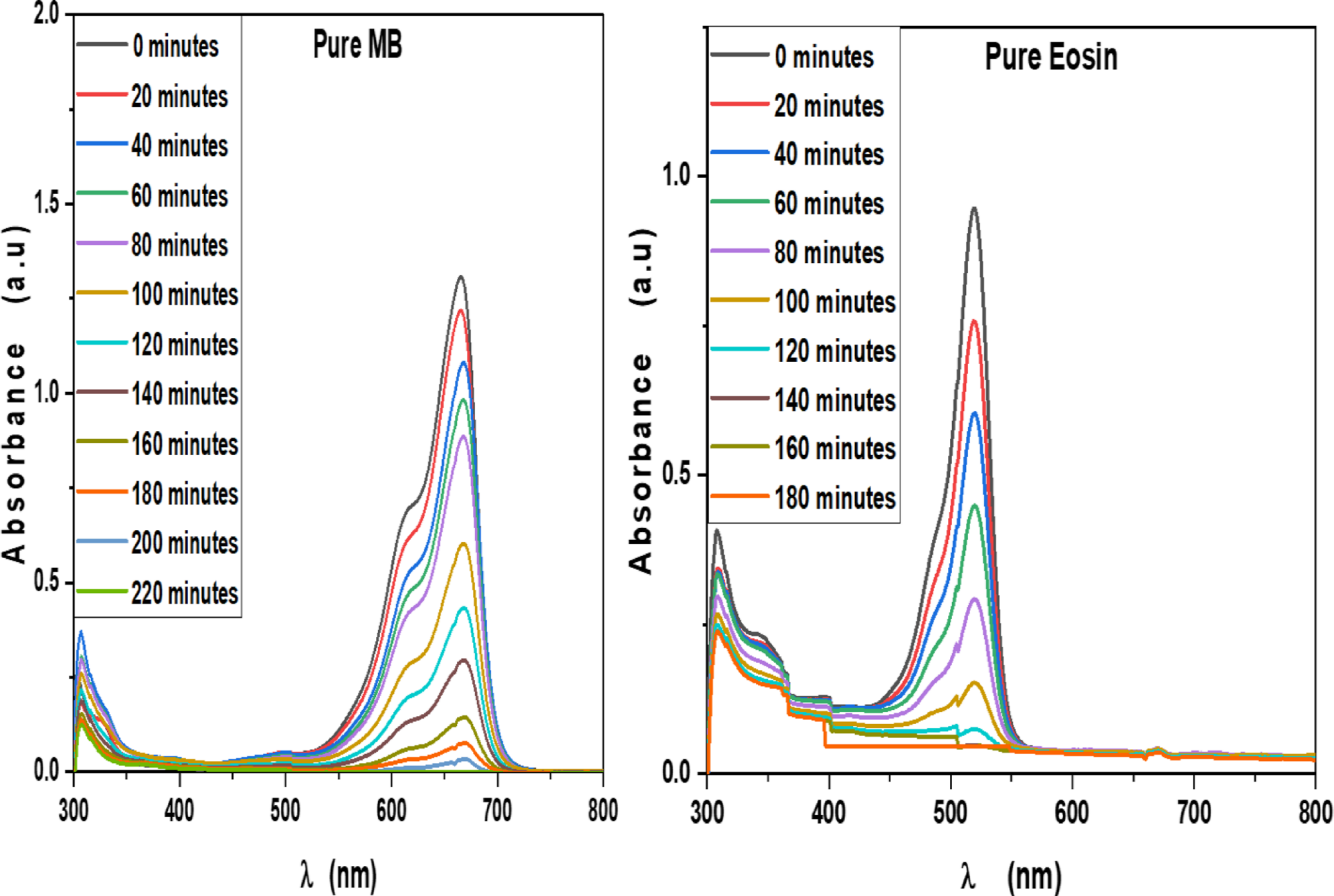
Absorbance spectra of pure Methylene blue dye solution and pure Eosin dye solution at different exposure times under solar light irradiation.

**Fig. 14:**
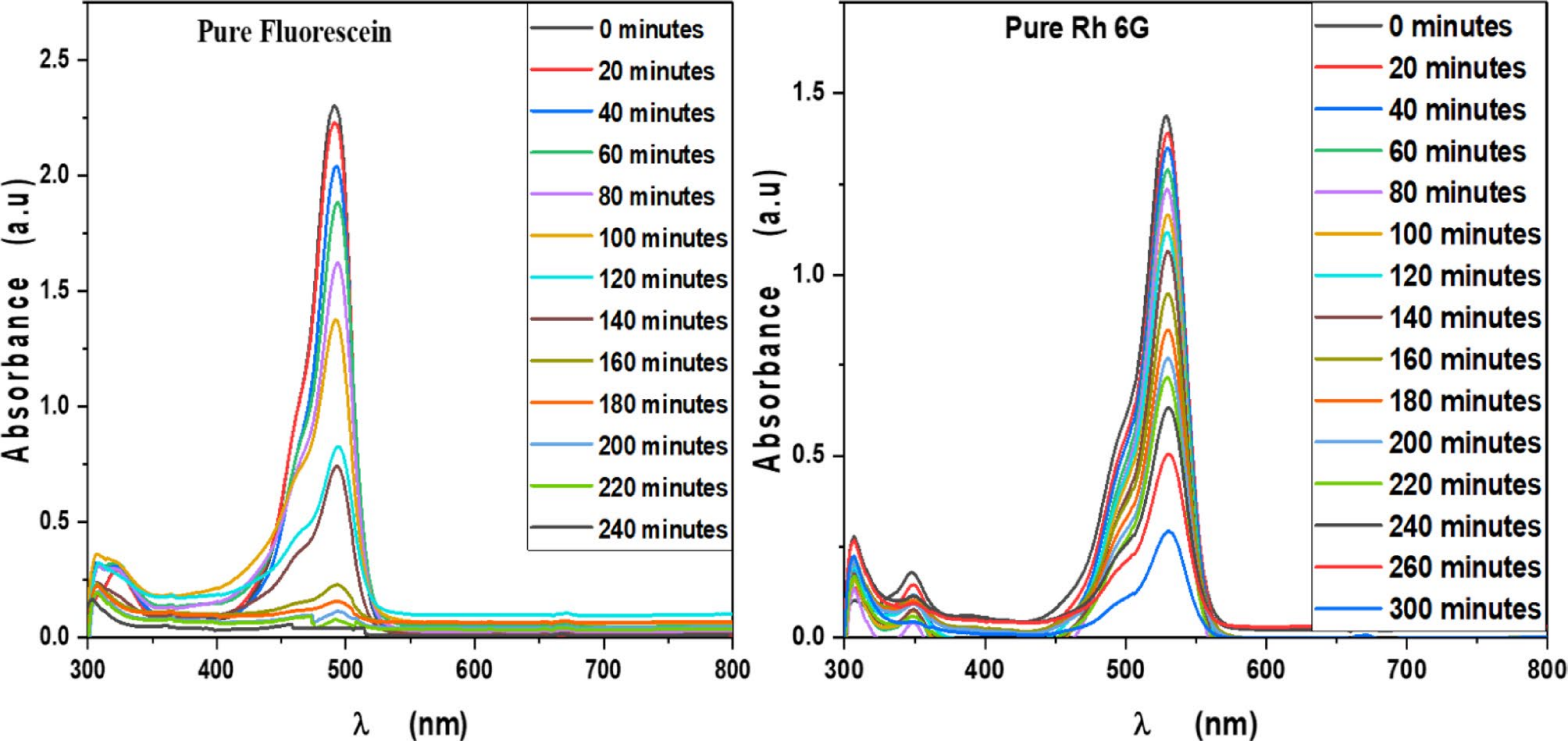
Absorbance spectra of pure Fluorescein dye solution and pure Rh 6G dye solution at different exposure times under solar light irradiation.

**Fig. 15:**
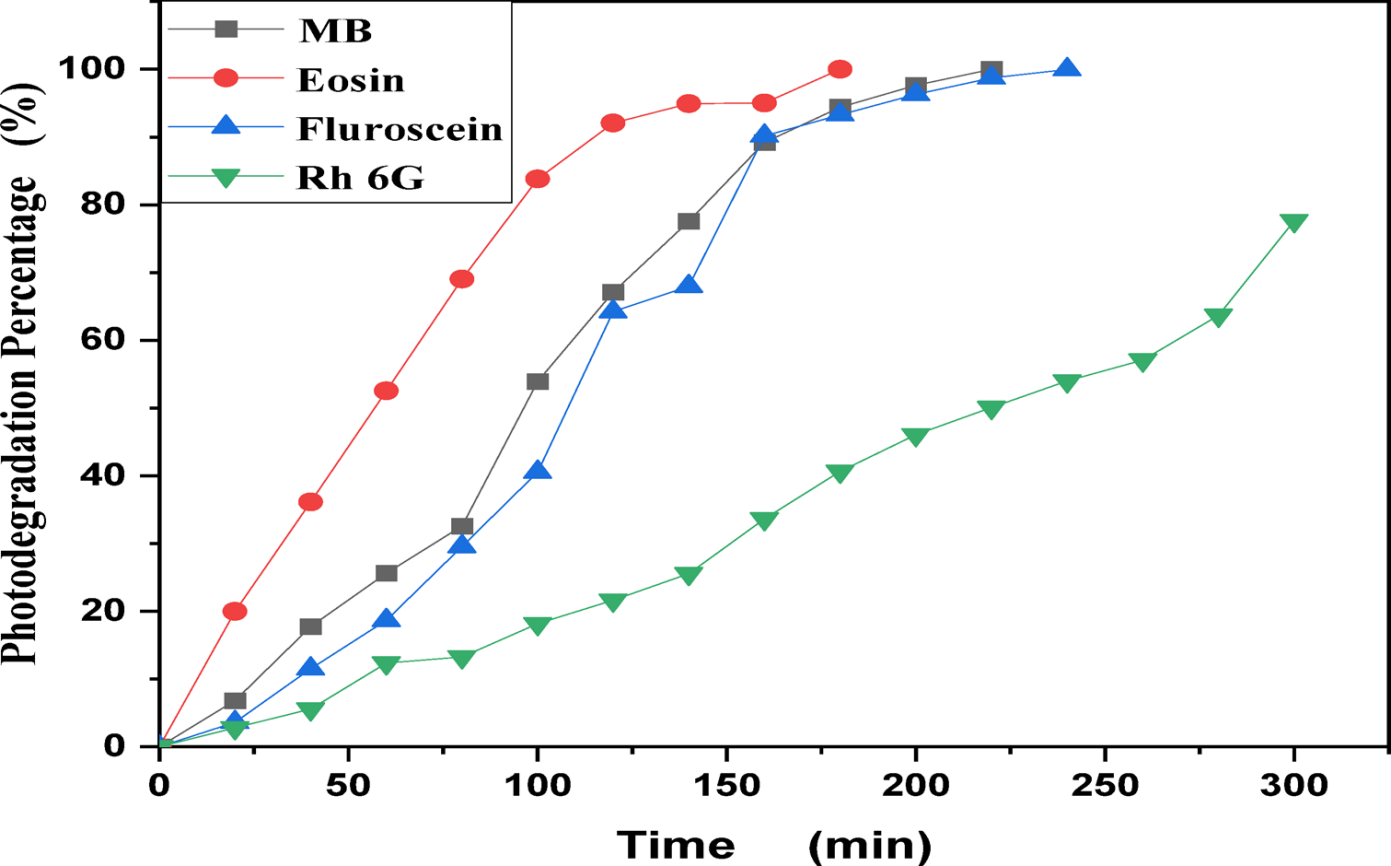
Percentage photodegradation of pure Methylene blue, pure Eosin, pure Fluorescein and pure Rhodamine 6G dye solutions vs. irradiation time.

**Fig. 16:**
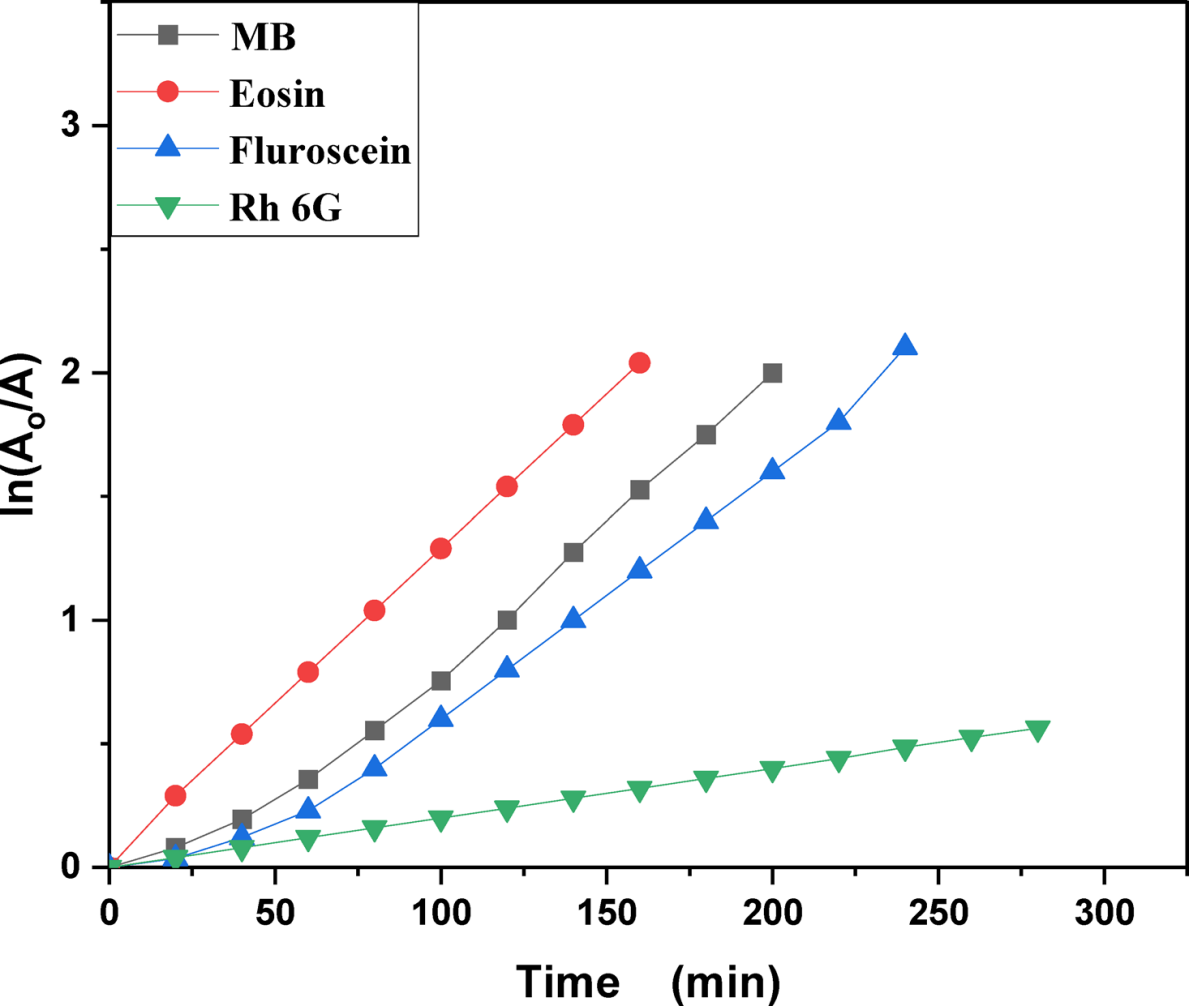
Kinetic plot of ln (A_o_/A) vs. irradiation time of pure Methylene blue, pure Eosin, pure Fluorescein and pure Rhodamine 6G dye solutions.

**Fig. 17:**
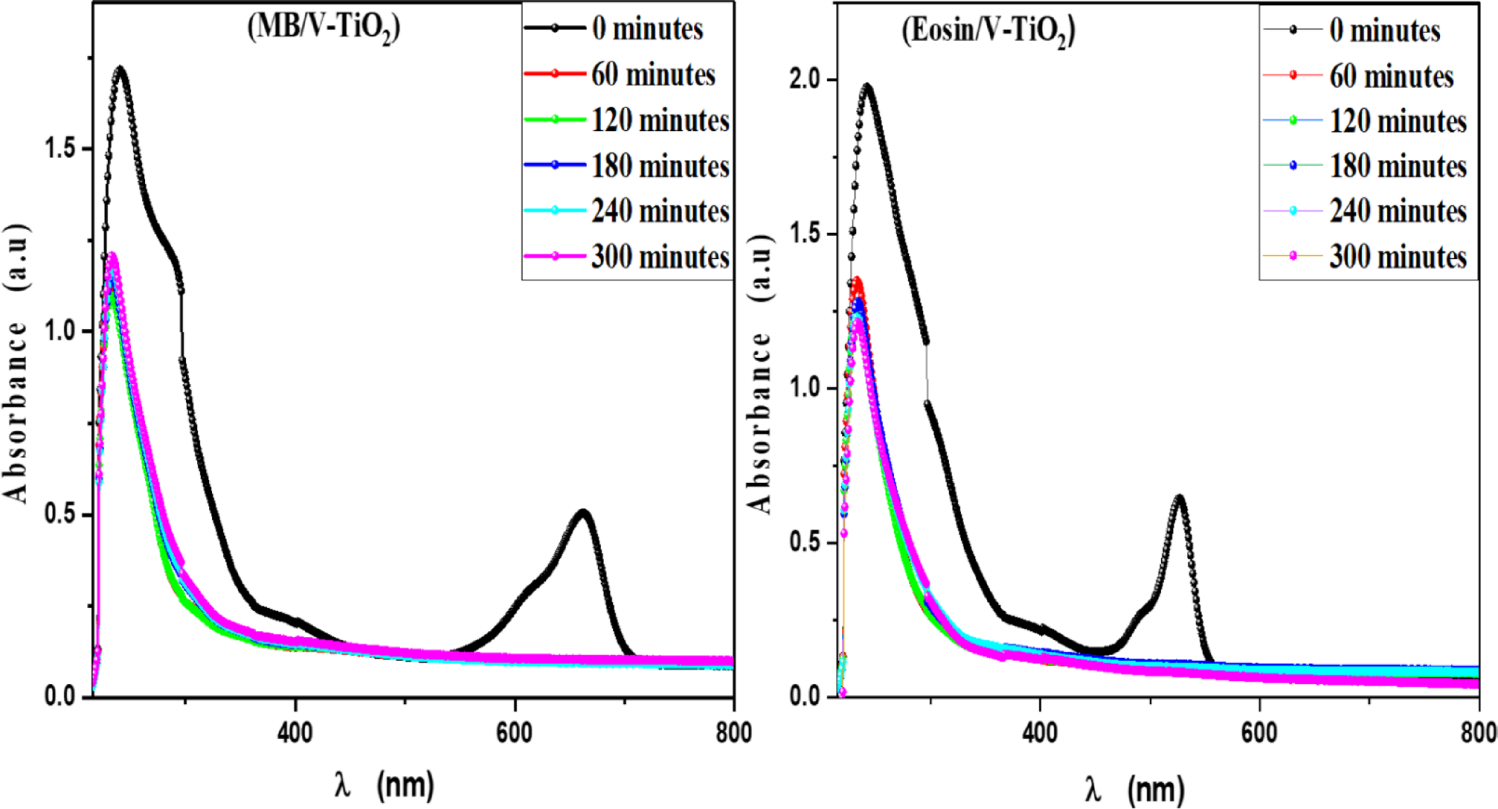
Absorbance spectra of a solid-state thin films of Methylene blue and Eosin dyes in the presence of the catalyst V-TiO_2_ NPs at different irradiation times.

**Fig. 18:**
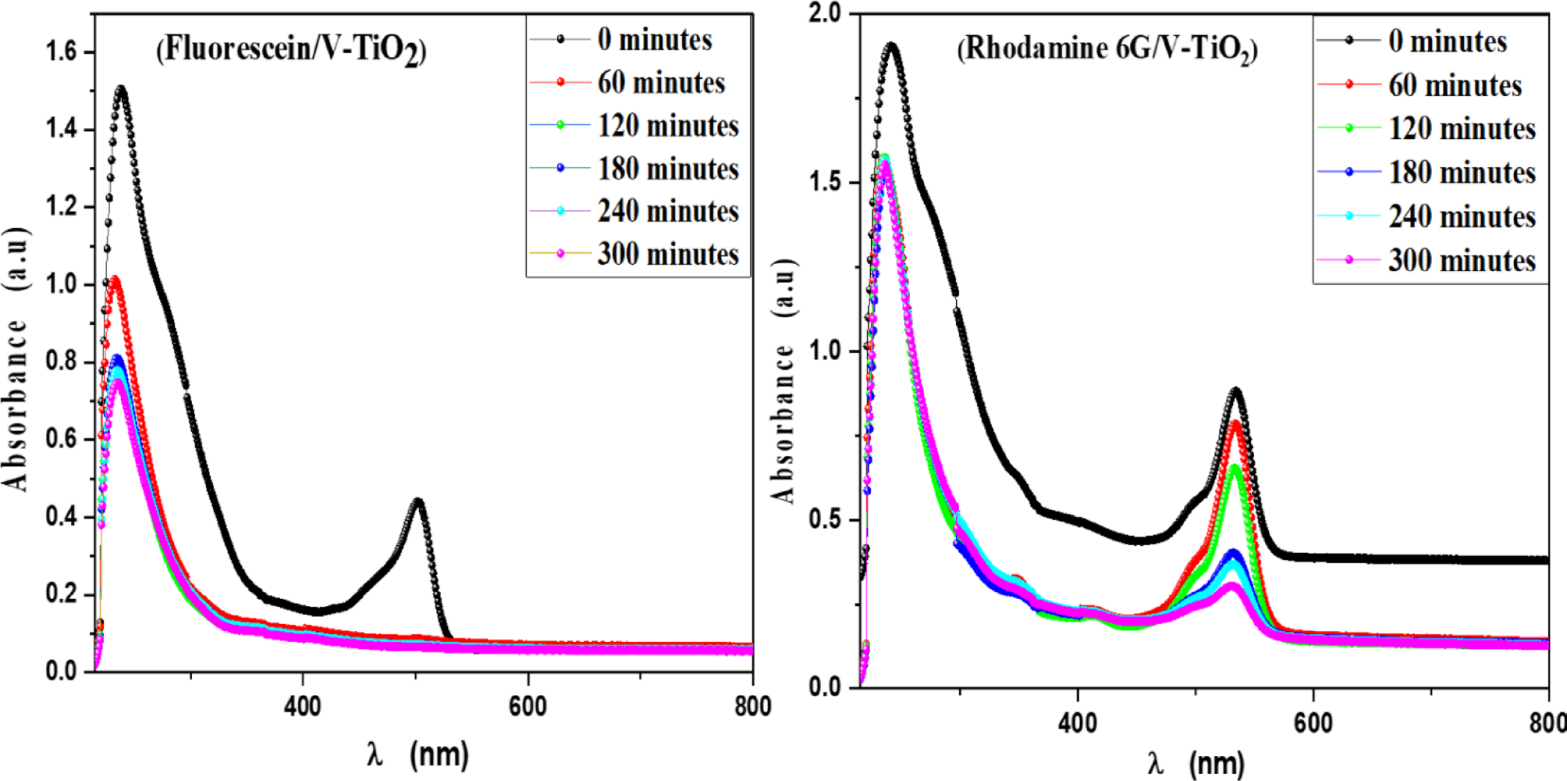
Absorbance spectra of a solid-state thin films of Fluorescein and Rhodamine 6G dyes in the presence of the catalyst V-TiO_2_ NPs at different irradiation times.

**Fig. 19:**
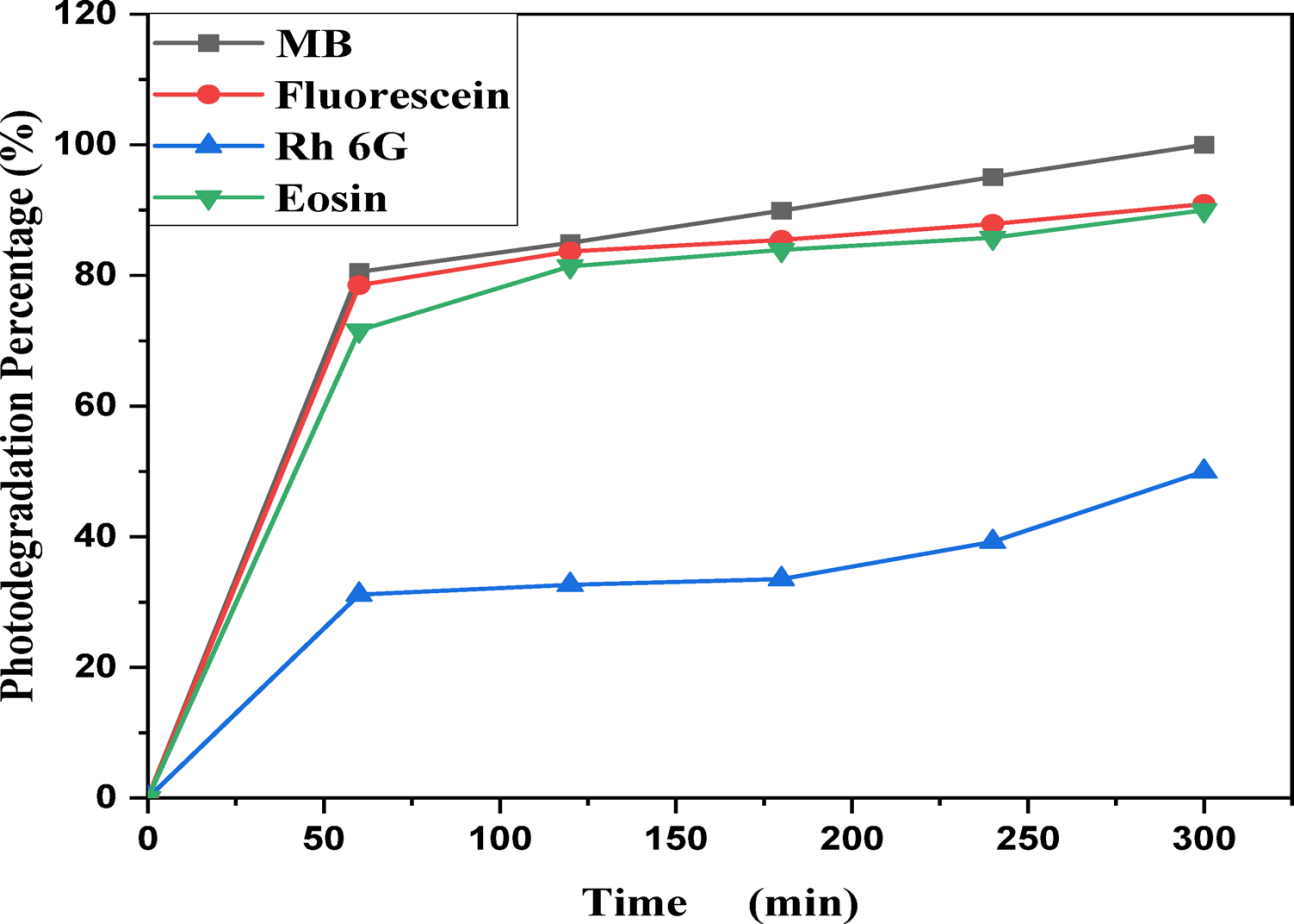
Percentage photodegradation of a solid-state thin film of a methylene blue, Eosin, Fluorescein and Rhodamine 6G dyes in the presence of the catalyst V-TiO_2_ NPs vs. irradiation time.

**Fig. 20:**
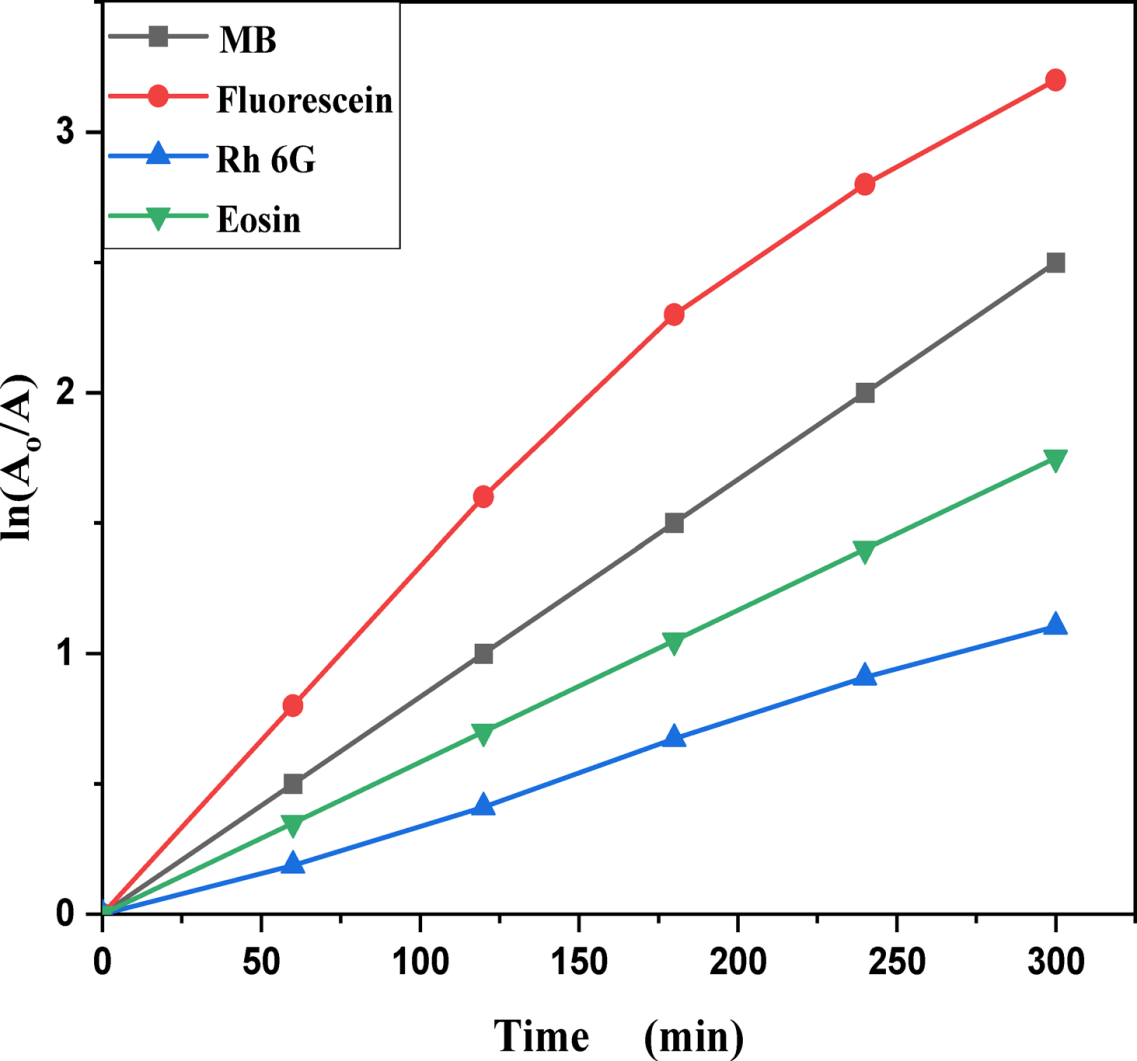
Kinetic plot of ln (A_o_/A) vs. irradiation time of a solid-state thin films of a methylene blue, Eosin, Fluorescein and Rhodamine 6G dyes in the presence of the catalyst V-TiO_2_ NP_s_.

**Fig. 21:**
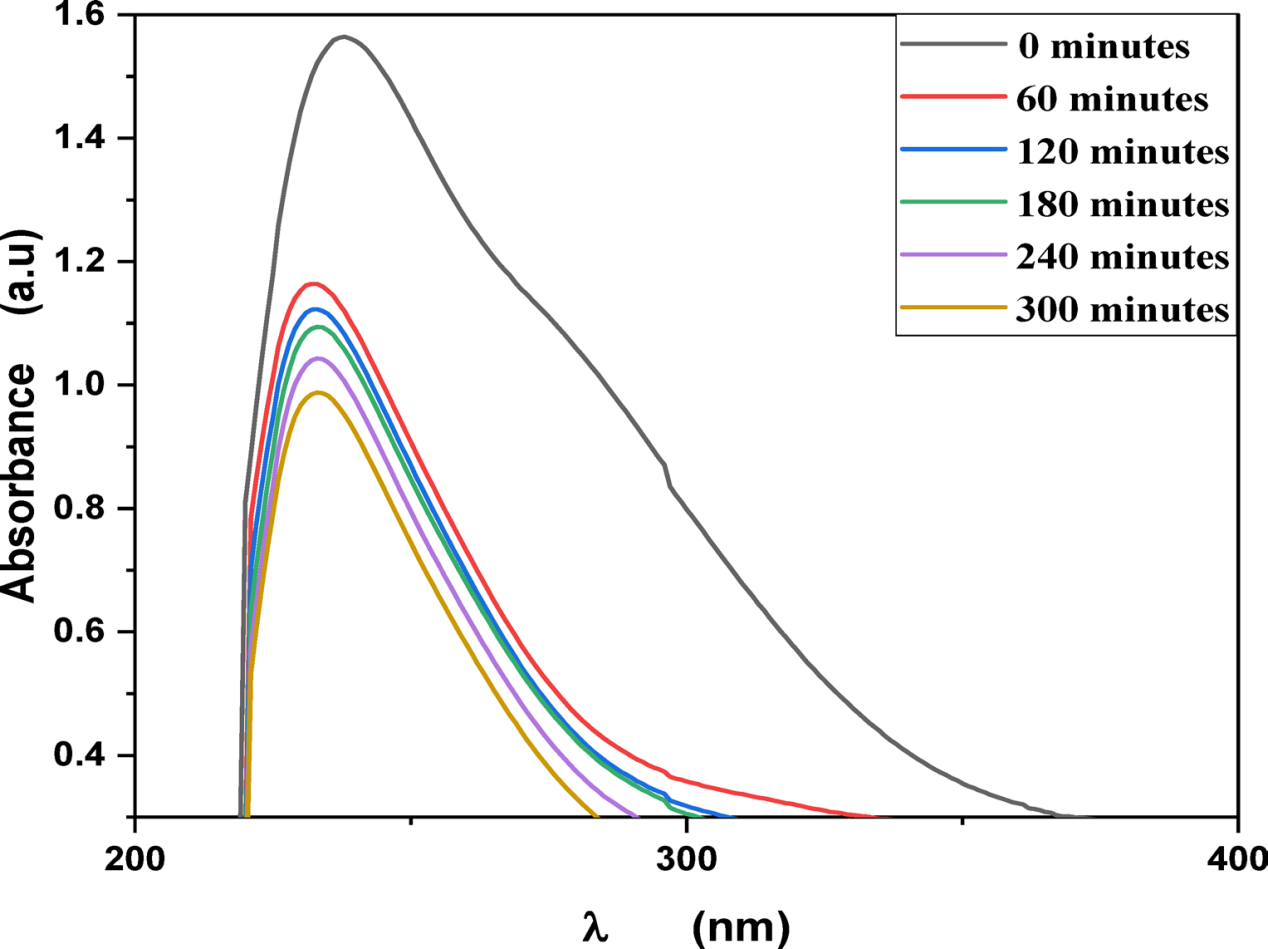
Absorbance spectra of the pure catalyst V-TiO_2_ NPs under solar light at different irradiation times.

MB, Eosin, Fluorescein and Rh 6G organic dyes were commonly found in a fabric wastes, were frequently used as a model pollutant in a wastewater treatment to evaluate the catalysts’ efficiency and used as a photosensitized nanomaterial in a dye sensitized solar cells. So, we were prepared a solutions of a pure MB, pure Eosin, pure Fluorescein and pure Rh 6G dye to study the photodegradation of these dyes under solar light irradiation. Where, Fig. 13 shows the optical absorbance spectra of pure MB dye solution and pure Eosin dye solution while Fig. 14 shows the optical absorbance of pure Fluorescein dye solution and pure Rh 6G dye solution under solar simulator light irradiation. Figures 13 and 14 showed that by increasing an irradiation time the MB, Eosin and Fluorescein dyes were rapidly photodegraded in a water but the Rh 6G dye was slowly photodegraded in a water under solar light irradiation due to the reduction of the optical absorbance of Rh 6G dye, the Rh 6G is a highly fluorescent synthetic dye and is widely used as a colored agent. Also, we were prepared a solid-state thin film of MB/V-TiO_2_ NP_s_, Eosin/V-TiO_2_ NP_s_, Fluorescein/V-TiO_2_ NP_s_ and Rh 6G/V-TiO_2_ NP_s_ for studying the effect of V-TiO_2_ NP_s_ as a catalyst on the photodegradation of a pollutant dyes. Figure 15 illustrates the temporal relationship between the photodegradation percentage (A_0_ − A/A_0_) and different irradiation time of pure MB, pure Eosin, pure Fluorescein and pure Rh 6G dyes and noticed that by increasing irradiation time the degradation percentage of pure dyes increases. Figure 16 illustrates the temporal relationship between the ln(A_0_/A) and irradiation time to determine the rate constant (K) and noticed that the rate constant was increased by increasing irradiation time of all pure dye solutions due to the rapidly photodegradation of dyes. Where A represents the optical absorbance of pure dye at time (t) of irradiation and A_0_ denotes its initial absorbance at zero minutes. As illustrated in Figs. 15 and 16, the photodegradation percentages of pure MB, pure Eosin, pure Fluorescein and pure Rh 6G dye solutions adhere to a pseudo-first-order rate equation, i.e., ln(A_0_/A) = Kt are shown in Table 4. The photocatalytic degradation for organic dye was observed utilizing the specific absorption peaks 664 nm for MB, 517 nm for Eosin, 533 nm for Rh 6G and 499 nm for Fluorescein) as a function of irradiation time over V-TiO_2_ NP_s_. Figure 17 shows the optical absorbance spectra of a MB/V-TiO_2_ solid-state thin film and Eosin/V-TiO_2_ solid state thin film, we were noticed that the λ_max_ of a MB dye in a solid-state thin film of V-TiO_2_ NP_s_ as a catalyst was gradually shifted by increasing a solar light irradiation time and the optical absorbance of a MB dye was decreased but the absorbance peak of V-TiO_2_ is slightly decreased under solar light irradiation. Also, Fig. 17 showed that the optical absorbance of an Eosin dyein a solid-state thin film was decreased in the presence of V-TiO_2_ NP_s_ as a catalyst by increasing a solar light irradiation time and the absorption peak of V-TiO_2_ was shifted to the blue region. Also, Fig. 18 shows the spectrum evolution of a Fluorescein/V-TiO_2_ solid-state thin film and Rh 6G/V-TiO_2_ solid state thin film. The optical absorbance of Fluorescein dye was decreased to be zero by increasing an irradiation time at 300 minutes and the absorption peak of V-TiO_2_ NP_s_ was shifted to the blue region. The absorbance of Rh 6G dye in a solid-state thin film of V-TiO_2_ NP_s_ as a catalyst was gradually decreased by increasing an irradiation time and the absorbance peak of V-TiO2 NP_s_ was decreased as shown in Fig. 18, but the Rh 6G dye is more stable to be degraded than other usable dyes. As reported by previous studies^39–43^ in the bulk solution, ^●^OH radicals primarily attack the aromatic chromophore ring, resulting in the breakdown of the Rh 6G structure and a decrease in absorbance without wavelength shift. Our results show that the Rh 6G is degraded slowly via the previously described mechanism. So, the V-TiO_2_ NP_s_ is the best catalyst to stabilize the Rh 6G dye to be applied in a dye sensitized solar cells. According to the above analysis, the prepared composite of V-TiO_2_ NP_s_ offers tremendous potential for wastewater treatment as an excellent photocatalyst in case of MB, Eosin and Fluorescein dyes. Additionally, the photocatalytic degradation rates of MB, Eosin, Fluorescein and Rh 6G over V-TiO_2_ NP_s_ were examined under solar light irradiation. Figure 19 illustrates the temporal relationship between the photodegradation percentage (A_0_-A/A_0_) and different irradiation time for solid-state thin films of MB, Eosin, Fluorescein and Rh 6G dyes with V-TiO_2_ NP_s_. Figure 20 illustrates the temporal relationship between the ln(A_0_/A) and irradiation time to determine the rate constant (K) and noticed that the rate constant was decreased by increasing irradiation time from 60 minutes to 300 minutes in case of Rh 6G but in case of MB, Eosin and Fluorescein, the rate constant become zero at irradiation times from 180 minutes to 300 minutes due to the completely degradation of dyes. Where A represents the concentration of MB at time (t) of irradiation and A_0_ denotes its initial concentration that shown in Table 5. Figure 21 shows the optical absorbance spectra (photostability) of the pure catalyst V-TiO_2_ NP_s_ under solar light irradiation at different exposure times from 60 minutes to 300 minutes, where the optical absorbance of V-TiO_2_ NP_s_ was slightly decreased by increasing irradiation time from 60 minutes to 300 minutes and indexed by a characteristic absorption peak of it.

**Table 4:** Photocatalytic degradation percentage and rate constant (K) for pure MB, pure Eosin, pure Rh 6G and pure fluorescein dye solutions under solar light irradiation.

Catalytic parameter		Rate constant (min^−1^) ± 0.0239					Degradation percentage (%)				
Time (minutes)		60	120	180	240	300	60	120	180	240	300
Dye	Fluorescein	0.0035	0.0086	0.015	0.0153	0.0156	18.68	64.25	93.29	99.52	99.98
	MB	0.0045	0.0093	0.016	0.0187	0.0192	25.63	67.10	94.42	99.85	99.91
	Eosin	0.0124	0.021	0.022	0.024	0.026	52.54	92.07	95.66	98.45	99.65
	Rh 6G	0.0017	0.0021	0.0029	0.0033	0.0036	12.36	21.63	40.66	54	77.67

**Table 5:** Photocatalytic degradation percentage and rate constant (K) for a solid-state thin film of MB, Eosin, Rh 6G and fluorescein dyes in the presence of V-TiO_2_ NP_s_ as a catalyst.

Catalyst	Rate constant (min^−1^) ± 0.0239							Degradation percentage (%)				
	Irradiation time (minutes)		60 min	120 min	180 min	240 mi ns	300 min	60 min	120 min	180 min	240 min	300 min
V-TiO_2_ NP_s_	Dye	Fluorescein	0.027	0.015	―	–	–	87.88	96.93	–	–	–
		Methylene Blue	0.027	0.014	–	–	–	90.56	99.3	–	–	–
		Eosin	0.022	0.013	–	–	–	89.98	98.98	–	–	–
		Rh 6G	0.004	0.003	0.0024	0.0021	0.001	31.15	32.62	33.50	39.23	50

### Photocatalytic reaction mechanism

**Fig. 22:**
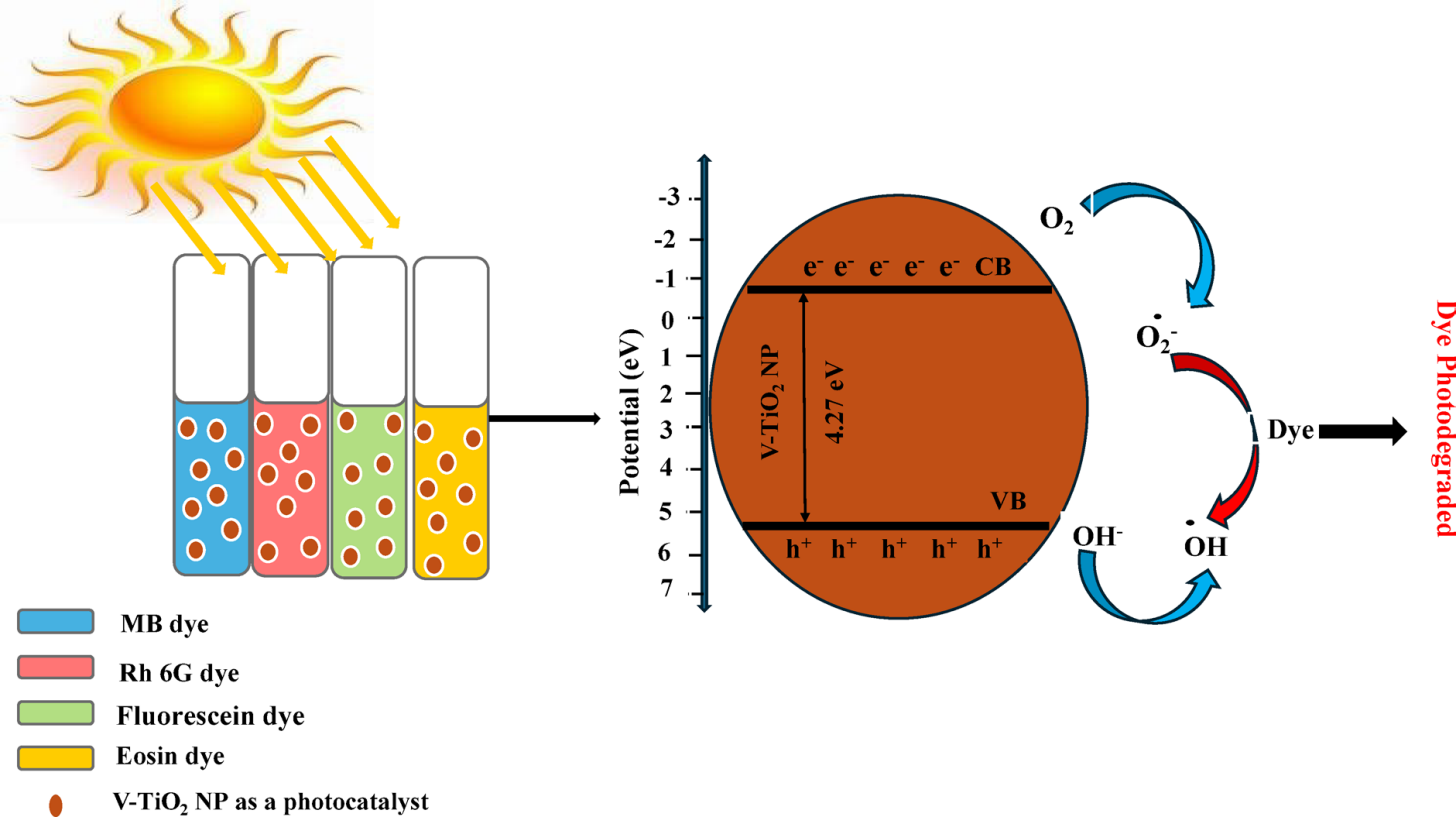
Photocatalytic Mechanism of the catalyst V-TiO_2_ NP_s_.

Based on the given experimental results and subsequent characterization studies, a possible photodegradation mechanism for different organic dyes over V-TiO_2_ NP_s_ is illustrated in Fig. 22. We can be explaining the charge-transfer process by calculating the photocatalytic activity conduction band minimum (CBM) and valence band maximum (VBM).

1 $${{\text{E}}_{{\text{CB}}}}={\text{ X}}--{{\text{E}}_{\text{e}}}--0.{\text{5}}{{\text{E}}_{\text{g}}}$$

2 $${{\text{E}}_{{\text{VB}}}}={\text{ }}{{\text{E}}_{\text{g}}}+{\text{ }}{{\text{E}}_{{\text{CB}}}}$$

E_VB_ and E_CB_ represent the valence and conduction band edge potentials, respectively. E_g_ represents the free electron energy of a semiconductor while E_e_ represents the standard hydrogen electrode (4.5 eV vs. NHE). The letter “X” denoted the geometric mean of the constituent elements’ absolute electronegativity^51^. The maximum valence band edge potential is −2.1 eV and the minimum conduction band edge potential is −6.37 eV. The excited electrons on the CB of V-TiO_2_ NP_s_ reduced O_2_ to ^●^O_2_^−^, While the holes in the VB were able to oxidize OH^−^ to produce ^●^OH then reacted with a dye to photodegraded. As shown in Figs. 17 and 18, when V-TiO_2_ NP_s_ (a scavenger of photogenerated electron (e^−^) is added, the photodegradation percentage of organic dyes increases compared to when no V-TiO_2_ NP_s_ is added as in Figs. 13 and 14. The main reason for this result is the efficient separation of photoinduced electron-hole pairs facilitated by the photoinduced electron consumption of V-TiO_2_ NP_s_. Figure 22 shows the photocatalytic mechanism of V-TiO_2_ NP_s_ based on the aforementioned experimental results. When solar light irradiates V-TiO_2_ NP_s_, electrons were excited from the VB to the CB, leaving the VB with holes. These photogenerated electrons and holes recombine slowly, resulting in effective electron-hole pair separation and increased photocatalytic performance. ^●^O_2_^−^ free radicals play a significant role in photocatalysis. Additionally, hydroxyl free radicals were generated in this system and attributed to photocatalytic degradation^24^.

## Conclusions

In summary, the catalyst vanadium doped titanium oxide nanoparticles were synthesized *via* a Sol-gel technique. The photodegradation of MB, Eosin, Fluorescein and Rh 6G organic dyes in the presence of V-TiO_2_ NPs was increased by increasing irradiation time under solar light. So, the V-TiO_2_ NPs as considered a good photocatalyst. The considerable enhancement in photocatalytic activity (~ 99.3% degradation of MB, ~ 96.51% degradation of Fluorescence, ~ 98.98% degradation of Eosin after 120 min and ~ 50% degradation of Rh 6G after 300 min) was ascribed to the critical role of Vanadium in the TiO_2_. Apart from the photocatalytic degradation activity, our work can greatly motivate the designing and synthesis strategies of other modified semiconducting nanomaterials. The vanadium doped titanium oxide nanoparticles is the best removal of dyes and can be applied as a pollutant removal in a wastewater application. Also, V-TiO_2_ NP_s_ with Rh 6G have successfully demonstrated the potential of enhancing the electrochemical performance of dye sensitized solar cells through the strategic adsorption Rhodamine 6G dye due to Rh 6G dye has a remarkably high photostability, high fluorescence quantum yields 0.95, has a fluorescence range of 550–590 nm, having a maximum fluorescence at 565 nm, its refractive index is 1.87 and k = 0.79 for 530 nm. It is often used as a tracer dye within water to determine the rate and direction of flow and transport.

## Data Availability

The supporting data of the current study are available from the corresponding author on reasonable request.
